# Early and opposing neutrophil and CD4 T cell responses shape pulmonary tuberculosis pathology

**DOI:** 10.1084/jem.20250161

**Published:** 2025-07-30

**Authors:** Benjamin H. Gern, Josepha M. Klas, Kimberly A. Foster, Molly E. Kanagy, Sara B. Cohen, Courtney R. Plumlee, Fergal J. Duffy, Maxwell L. Neal, Mehnaz Halima, Andrew T. Gustin, Sylvia M. Stull, Jasmine J. Wilson, Alan H. Diercks, Alan Aderem, Michael Gale, John D. Aitchison, Michael Y. Gerner, Kevin B. Urdahl

**Affiliations:** 1 Center for Global Infectious Disease Research, Seattle Children’s Research Institute, Seattle, WA, USA; 2 Department of Pediatrics, University of Washington, Seattle, WA, USA; 3 Department of Global Health, University of Washington, Seattle, WA, USA; 4 Department of Immunology, University of Washington, Seattle, WA, USA

## Abstract

Pulmonary *Mycobacterium tuberculosis* (Mtb) infection results in a variety of heterogeneous lesion structures, from necrotic granulomas to alveolitis, but the mechanisms regulating their development remain unclear. Using a mouse model of concomitant immunity and subsequent aerosol infection, we demonstrate that counter regulation between neutrophils and CD4 T cells occurs very early during infection and governs these distinct pathologies. In primary Mtb infection, a dysregulated feed-forward circuit of neutrophil recruitment occurs, in which neutrophils hinder CD4 T cell interactions with infected macrophages, cause granuloma necrosis, and establish a replicative niche that drives a two-log increase in lung bacterial burden. Conversely, the rapid recruitment and activation of T cells due to concomitant immunity promotes local macrophage activation and dampens detrimental neutrophil responses. Together, these studies uncover fundamental determinants of tuberculosis lung pathology, which have important implications for new strategies to prevent or treat tuberculosis.

## Teaser

Gern et al. show that antagonistic circuits between neutrophils and CD4 T cells are established during early Mtb infection, and the relative kinetics of these responses determines the pathologic outcome of infection.

## Graphical Abstract

**Figure:**
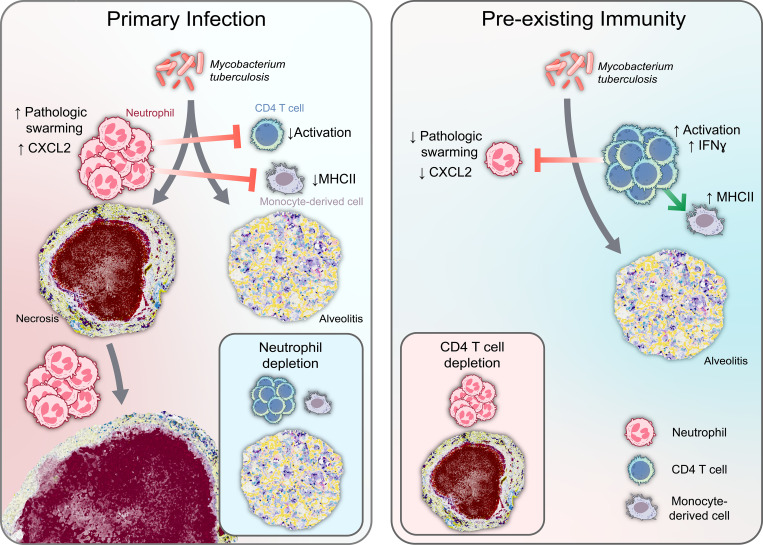

## Introduction

The outcomes of aerosol infection with *Mycobacterium tuberculosis* (Mtb), the bacteria that causes tuberculosis (TB), are highly heterogeneous and shaped by prior immunity, including immunity from vaccination or prior Mtb exposure (Hunter, 2016). The pulmonary granuloma, an organized aggregate of immune cells, often with a necrotic core that destroys normal lung architecture, is frequently considered the hallmark lesion of TB (Pagán and Ramakrishnan, 2015). However, human postmortem studies in the pre-antibiotic era showed that many Mtb-infected lung lesions do not exhibit granulomatous architecture (Rich, 1944). In primary TB, where patients had no previous exposure to Mtb, pulmonary lesions usually start as granulomas, with a core of macrophages that often undergo necrosis centrally surrounded by a lymphocytic cuff. Conversely in after primary TB (when individuals had prior Mtb exposure), early lesions are often comprised of pneumonia-like alveolitis, with infected cells contained within intact alveolar sacs (Hunter, 2016). Despite appreciation of the association between prior immunity and Mtb lesion types for more than a century, the mechanisms by which prior immunity promotes the development of alveolitis instead of granulomas remain unknown.

In modern times, human postmortem studies are rare, and most research dissecting TB immunity is performed in animals without prior Mtb exposure. These studies have revealed many insights about the varied microenvironments within granulomas that restrict immune function, including distinct myeloid cell niches (macrophage subtypes, monocytes, and granulocytes) and various factors that suppress T cell effector functions. T cells are frequently relegated to the peripheral cuff, being unable to infiltrate the granuloma cores and engage in cognate interactions with infected cells (Gautam et al., 2017; Kauffman et al., 2017). Lesions also directly suppress T cells through local immunoregulatory factors, including TGFβ (Gern et al., 2021) and products of tryptophan metabolism (Gautam et al., 2017), some of which are spatially partitioned in distinct immunoregulatory domains, leading to localized immune suppression (McCaffrey et al., 2022). It is largely unknown how these microenvironments and immune regulatory factors differ in granulomatous versus alveolitis lesions, raising the possibility that host-directed therapies may be effective only in certain lesion types. Furthermore, lung-destructive necrotic lesions can progress to cavitary TB disease, which takes longer to respond to antibiotic treatment, has a high risk of relapse, and can lead to significant morbidity from posttuberculous lung disease. Thus, understanding how to prevent this destructive pathology from developing could lead to new strategies to curb severe manifestations of disease (Allwood et al., 2021; Hernandez-Romieu et al., 2019; Lewinsohn et al., 2017; Ravimohan et al., 2018; Benator et al., 2002).

Historically, mouse models have lacked the ability to dissect relationships between lesion structure and disease control. Mtb-infected C57BL/6 mice, the most commonly used mouse strain for TB research due to the abundance of tools for mechanistic studies, generate diffuse lung inflammation and not discrete granulomas when infected with a conventional aerosol dose of 50–100 CFUs (Kramnik and Beamer, 2016). This has restricted most mechanistic studies to enumeration of lung bacterial burden as the sole outcome and caused heavy scrutiny of the model. Reducing the infectious dose to a more physiologic 1–3 CFUs was shown to induce formation of well-circumscribed lesions that share properties with stereotypical human lesions, including discrete and segregated regions containing T cells and infected macrophages, yet these lesions still lack necrotic cores and do not fully recapitulate diverse forms of human disease (Plumlee et al., 2021). In contrast, C3HeB/FeJ (C3H) mice do develop necrotizing granulomas, especially when infected with hypervirulent Mtb strains of the W-Beijing lineage (Moreira-Teixeira et al., 2020b; Cohen et al., 2024). A single gene, *Sp140*, has been identified that confers the extreme susceptibility and necrotic lesions of C3H mice (Ghiboub et al., 2022), and *Sp140*^−/−^ mice on a C57BL/6 background exhibit similar elevated bacterial burdens and necrotic lung pathology as C3H mice (Ji et al., 2021). Use of these mouse models has led to the identification of critical signaling pathways that regulate Mtb infection outcomes, including type I IFN versus IL-1, as well as insights into the temporal processes driving inflammation and disease: neutrophil recruitment, cellular death, plasmacytoid dendritic cell sensing, and IFN production/signaling (Ji et al., 2021; Kotov et al., 2023; Moreira-Teixeira et al., 2020a). Additional work using collaborative cross mice has shown that host genetics can heavily influence disease susceptibility and ability to control Mtb after immunization with bacillus Calmette–Guérin (BCG) (Lai et al., 2023; Smith et al., 2022; Smith et al., 2016). Protection as measured by bacterial burden in these models is associated with differences in T cell effector responses and concordant structural changes of pulmonary lesions. Together, these studies suggest a complex interplay between host and pathogen genetics prior Mtb exposure history and local immune function and downstream disease outcome.

Here, we coupled the C3H mouse model with a model of concomitant immunity to examine the factors regulating the formation of two divergent types of TB lesions: necrotizing granulomas versus alveolitis, based on prior exposure history. We show that during primary Mtb infection, neutrophils dominantly and detrimentally shape the landscape of pathology by suppressing CD4 T cell and macrophage activation, providing an early niche for Mtb replication, and driving both the generation and propagation of centralized lesion necrosis. In contrast, concomitant immunity elicits early T cell recruitment to sites of infection, where CD4 T cells induce macrophage activation and disrupt neutrophil-driven lung pathology to elicit formation of alveolitis. This counter regulation results in long-lasting changes in granuloma structure, even at time points of CFU convergence, indicating a decoupling of bacterial burden from pathological outcomes. Together, our findings uncover key immunological mechanisms underlying divergent tissue pathologies during Mtb infection and provide critical insights into development of novel strategies to shape lesion structure and treat TB.

## Results

### Pre-existing immunity abrogates the formation of necrotic granulomas

**Figure 1.**
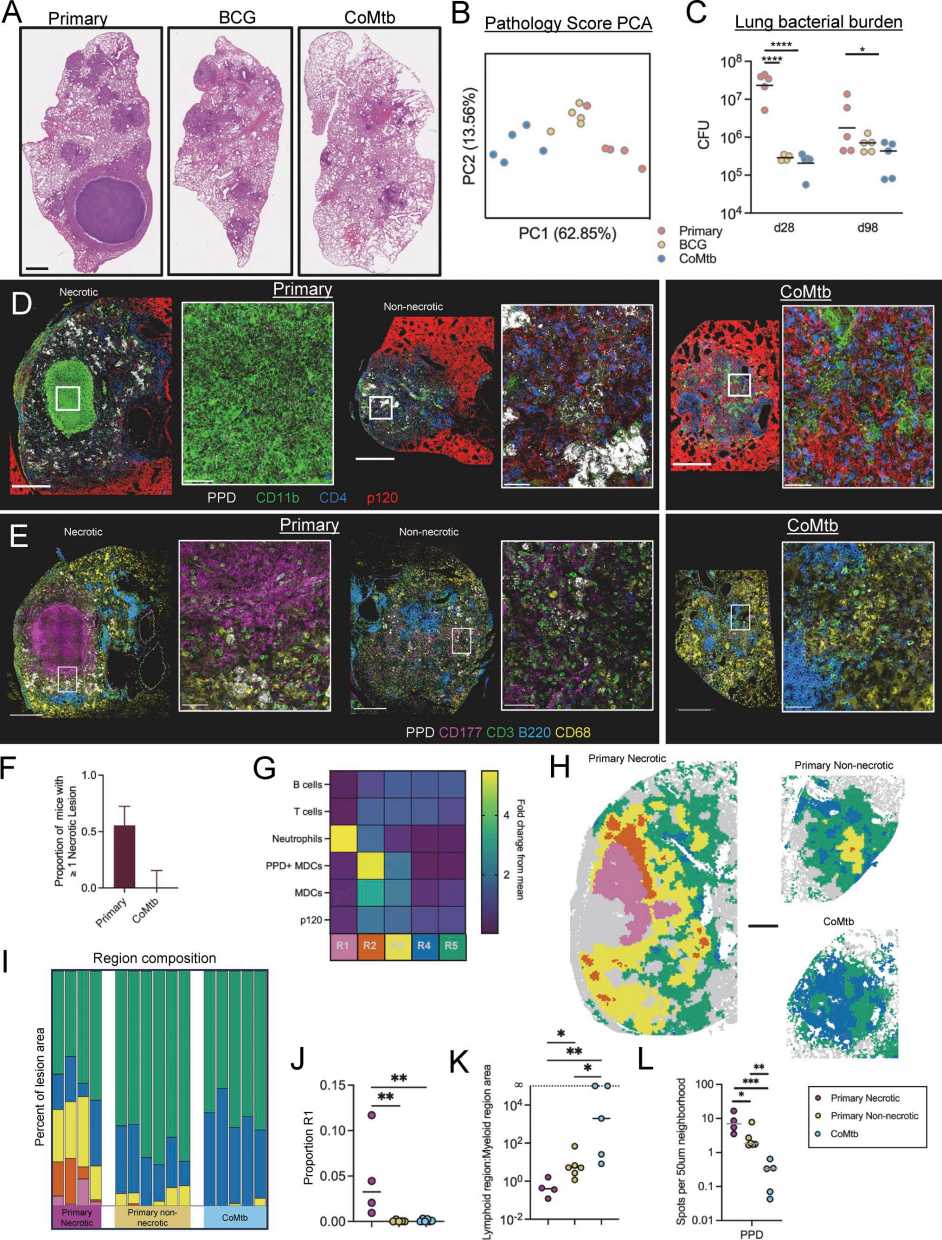
**Pre-existing immunity abrogates the formation of necrotic granulomas. (A–C)** d98 after CD infection (*n* = 5 per group). **(A)** Representative histology images of lung sections. Scale bar: 1 mm. **(B)** PCA of pathology scores. **(C)** Mtb lung burden in primary, BCG, and CoMtb groups. **(D–L)** d35 after ULD infection (*n*= 10 primary, 5 CoMtb). **(D)** Representative confocal microscopy images demonstrating preserved alveolar integrity in non-necrotic primary and CoMtb lesions. Scale bar: 500 μm; zoom: 50 μm. **(E)** Representative confocal microscopy images depicting major cell populations within lesions. Scale bar: 500 μm; zoom: 50 μm. **(F)** Percent of mice with necrotic lesions, covers two independent experiments. **(G)** Heatmap showing cellular composition of clustered microenvironments. **(H)** Representative map showing 50 µm neighborhoods, color-coded microenvironment. Scale bar: 500 μm. **(I)** Percent area of lesion comprised by each microenvironment. Uninvolved regions (gray) not included. **(J)** Percent of lesion comprised by necrotic region (pink). **(K)** Ratio of lymphoid (blue and green) to myeloid (yellow, orange, and pink) predominant regions. **(L)** Relative density of PPD signal per 50 µm^2^ neighborhood. Single-group comparisons by Mann–Whitney U test. *P < 0.05, **P < 0.01, ***P < 0.001, ****P < 0.0001, and ns, P ≥ 0.05. Error bars (F) reflect 95% confidence intervals. Points represent individual mice or lesions from individual mice. Data are representative of one (A–C) or two (D–L) independent experiments with at least four mice per group. See also Fig. S1. PCoA, principal coordinate analysis

**Figure S1.**
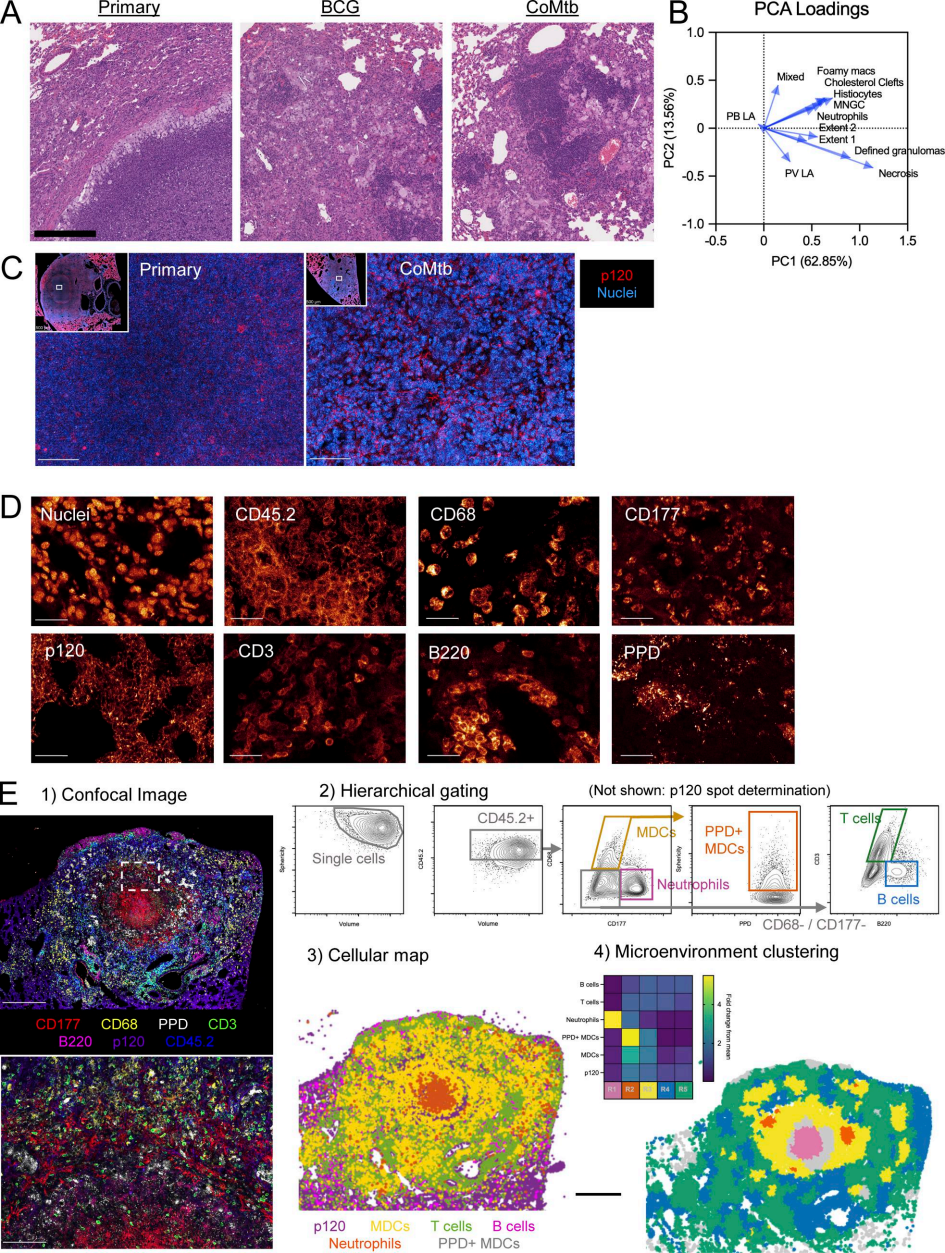
**Related to** Fig. 1 **.** Pre-existing immunity abrogates the formation of necrotic granulomas: **(A)** Lesion zoom-ins from Fig. 1 A. Scale bar: 1 mm. **(B)** PCA loadings from Fig. 1 B. **(C)** Representative confocal images showing necrotic debris in center of primary necrotic lesion and intact nuclei surrounded by p120 staining within lesion formed in setting of CoMtb. Scale bar: 50 μm. **(D)** Representative confocal images of markers used for analysis in Fig. 1. Scale bar: 25 μm. **(E)** Histo-cytometry gating scheme to determine cell types for analysis in Fig. 1, G–K. Scale bar: 500 μm; zoom: 100 μm.

To examine the impact of pre-existing or ongoing immune responses against Mtb on de novo lesion structure development and disease progression, we utilized the C3H mouse model, which generates large necrotic granulomas akin to those found in human primary TB, especially after aerosol infection with hypervirulent or high transmission W-Beijing Mtb strains (Moreira-Teixeira et al., 2020b; Verma et al., 2019; Cohen et al., 2024). To induce pre-existing or concomitant immunity to Mtb, we used two established modalities: subcutaneous BCG immunization, as well as concomitant Mtb infection (CoMtb), in which a low-level chronic Mtb infection is established in the cutaneous lymph node after intradermal Mtb inoculation, without substantial dissemination to systemic sites (Kupz et al., 2016; Nemeth et al., 2020). Mice administered either BCG or CoMtb or unimmunized controls (primary infection) were aerosol infected 8 wk later with a conventional dose (CD, 50–100 CFUs) of SA161 Mtb, a hypervirulent clinical isolate from the W-Beijing lineage. As expected, when assessed at day 98 (d98) postinfection (p.i.), unimmunized mice developed large granulomas with a central necrotic core rimmed by foamy macrophages, surrounded by a lymphocytic cuff, as well as multiple smaller lesions without overt necrosis (Fig. 1 A and Fig. S1 A). In stark contrast, both CoMtb and BCG completely blocked the formation of necrotic granulomas, instead inducing smaller, less-organized lesions comprised of histiocytes and lymphoid cells, and exhibiting less neutrophil infiltration (Fig. 1 A and Fig. S1 A). Blinded, quantitative evaluation of multiple lung pathology metrics using principal component analysis (PCA) showed that CoMtb resulted in the greatest overall changes in pathology as compared with primary Mtb-infected mice (Fig. 1 B, Fig. S1 B, and Table S1). Both BCG and CoMtb markedly reduced bacterial burdens at d28 p.i., together indicating that pre-existing and concomitant immunity offer robust protection in this metric at early time points. Despite this and the stark differences in pathology at d98, differences in lung bacterial burdens were less pronounced 98 days p.i. and only significantly lower in the CoMtb group (Fig. 1 C), suggesting that protection from destructive lung pathology may be regulated independently from protection measured by bacterial burdens. Given the improved outcomes seen with CoMtb as compared with BCG, we chose CoMtb as the modality of prior immunity to dissect mechanistically.

Detection of multiple distinct lesion types in the same animal after primary infection, including both necrotic and non-necrotic lesions, raised the question of whether this reflected distinct stages of lesion progression (i.e., initial aerosol-seeded versus secondary, disseminated lesions) or an earlier divergence in lesion organization. To test these possibilities, we utilized an ultra-low dose (ULD) aerosol infection (1–3 CFUs), which results in the formation of a solitary organized lesion in most mice (Plumlee et al., 2021). Primary and CoMtb mice were assessed at d35 p.i., a time point shortly after the formation of mature lesions. In primary animals, we observed formation of single lesions in 27/40 infected mice across two experiments. These lesions possessed heterogeneous organization, with 15/27 of these single lesions presenting as granulomas containing a central necrotic core dominantly comprised of neutrophils (CD177-positive cells), necrotic debris (nuclear dye), and absence of alveolar epithelial staining (p120), consistent with destruction of the epithelial architecture (Fig. 1, D–F and Fig. S1 C). In stark contrast, the remaining 12/27 solitary lesions in primary infected mice lacked this necrotic core and instead contained tightly aggregated clusters of antigen-bearing macrophages (CD68^+^, purified protein derivative [PPD]+), which were surrounded by intact alveolar epithelium (Fig. 1, D–F), consistent with alveolitis.

We next examined early lesions after ULD Mtb infection of mice with CoMtb infection. We observed complete absence of lesion necrosis, and instead these lesions again were comprised of tightly aggregated infected macrophages surrounded by intact alveolar epithelium, consistent with alveolitis. To quantify these findings, we used histo-cytometry and CytoMAP (Stoltzfus et al., 2020; Gerner et al., 2012). We first segmented single cells to define major cell types within imaged tissues (neutrophils [CD177], macrophages [CD68], T cells [CD3 and CD4], B cells [B220], and Mtb antigen-bearing cells [PPD]) and also examined alveolar epithelial integrity with p120 staining (Fig. S1 D). We next used CytoMAP to raster scan the spatial neighborhoods (radius = 50 μm) within the imaging data and clustered these neighborhoods into discrete tissue region subtypes (i.e., microenvironments) based on the similarity of cellular composition (Fig. S1 E). Analysis of single lesions from one experiment revealed that 4/10 lesions in the setting of primary infection had regions consistent with necrosis (neutrophil enrichment, paucity of alveolar epithelium, R1/pink) and high antigen abundance (R2/orange), surrounded by regions of high myeloid density (R3/yellow) (Fig. 1, G–J). In contrast, lesions in CoMtb mice were comprised of regions with intact p120 staining and were highly enriched for lymphoid-dominant regions (R4/blue, R5/green), consistent with alveolitis (Fig. 1, I and K). Necrotic granulomas in primary animals were also associated with increased PPD abundance as compared with non-necrotic lesions, while lesions in CoMtb mice had markedly reduced PPD abundance (Fig. 1 L), consistent with markedly decreased CFU at early time points (Fig. 1 C). The structural heterogeneity in solitary lesions revealed by the ULD infection model indicated that there is an early divergence in primary lesion development (necrosis versus alveolitis) even in genetically identical C3H mice infected with the same Mtb strain, and that concomitant immunity afforded by CoMtb abrogates formation of necrotic granulomas and leads to the generation of alveolitis.

### CoMtb alters the immune landscape following Mtb infection

**Figure 2.**
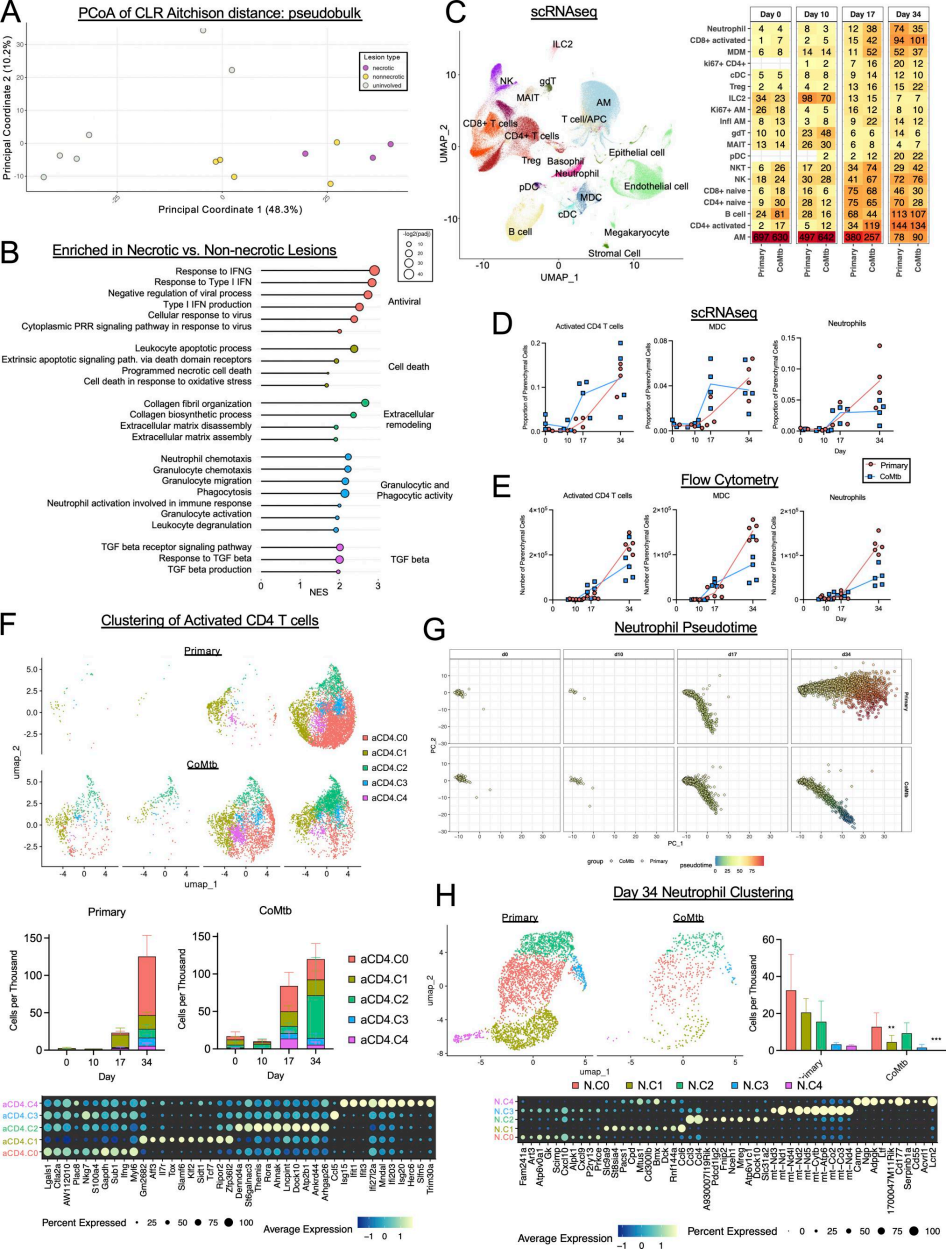
**CoMtb alters the immune landscape following Mtb infection. (A and B)** d35 after ULD infection, *n* = 8. **(A)** PCoA analysis of ROI transcriptomes, colored by lesion type (necrotic versus non-necrotic, 3 ROIs per point) or location (uninvolved, 1 ROI per point). **(B)** GSEA analysis showing enriched pathways per lesion type. **(C–E)** Multiple time points after CD infection. **(C)** UMAP depicting cell types identified by scRNAseq analysis of lung parenchymal cells, heatmap showing cellular abundance, numbers reflect the median number of cells per thousand. **(D)** Proportions of selected cell populations over time, scRNAseq. **(E)** Numbers of selected cell populations over time, flow cytometry. **(F)** UMAP depicting clusters within activated CD4^+^ cell cluster from C, heatmap showing the top 10 discriminatory genes. **(G)** Pseudotime analysis showing neutrophils across time points. **(H)** UMAP depicting clusters within d34 neutrophils from C, as well as heatmap showing the top 10 discriminatory genes. Points represent individual lesions (A, 3 ROIs samples per lesion, 1 per uninvolved area) and individual mice (D–F). False discovery rate–adjusted P values determined using the R fgsea package. Single-group comparisons by *t* test. Data are representative of one (A–D and F–H) or two (E) independent experiments with at least four mice per group. See also Fig. S2. UMAP, uniform manifold approximation and projection.

**Figure S2.**
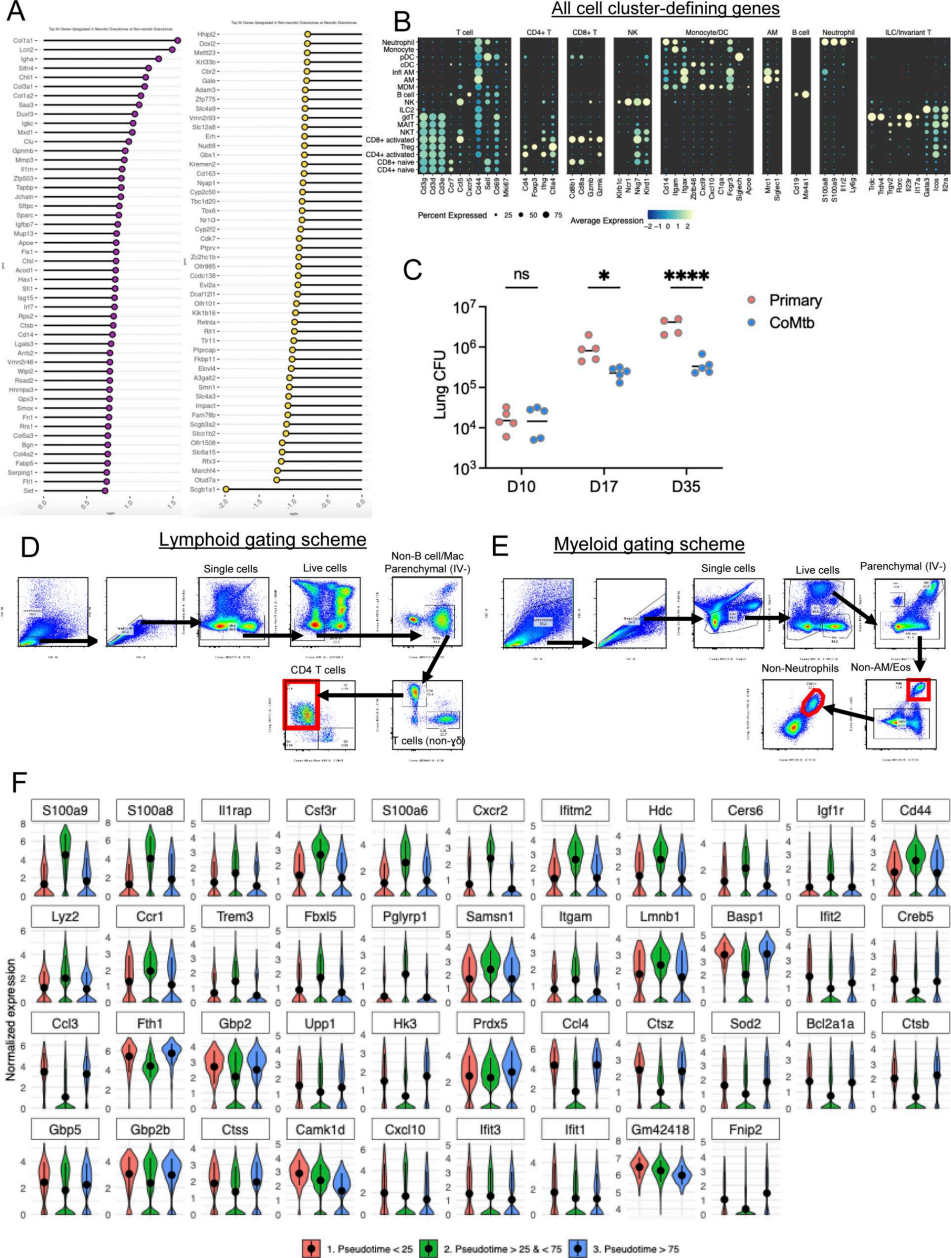
**Related to** Fig. 2 **.** CoMtb alters the immune landscape following Mtb infection. **(A)** Top 50 differentially expressed genes for necrotic and non-necrotic lesions in Fig. 2 A. **(B)** Top genes that discriminate scRNAseq clustering into the specific cell types in Fig. 2 C. **(C)** Pulmonary bacterial burdens corresponding to Fig. 2 E. **(D)** Lymphoid flow cytometry gating scheme for Fig. 2 E. **(E)** Myeloid flow cytometry gating scheme for Fig. 2 E. **(F)** Neutrophil pseudotime analysis showing differentially expressed genes within low (<25), intermediate (25–75), and high (>75) groups. Single-group comparisons by unpaired *t* test. *P < 0.05, ****P < 0.0001, and ns, P ≧ 0.05. Correlations by Pearson’s correlation test.

We next sought to obtain a more holistic understanding of lesion divergence at early time points. For this, we performed spatial transcriptomics analysis using the Nanostring GeoMx platform on necrotic and non-necrotic lesions from primary ULD-infected mice (d35 p.i.). We selected multiple regions of interest (ROIs) within necrotic and non-necrotic lesions, as well as from uninvolved distal lung regions. ROI counts for each lesion, and uninvolved region were aggregated, normalized, and assessed by PCoA. This analysis revealed major transcriptomic distinctions between lesions and uninvolved tissues. Further, while ROIs from non-necrotic lesions were dispersed along PCoA 1 and had a range, which overlapped with that of uninvolved tissues, ROIs from necrotic granulomas were tightly clustered and entirely distinct from uninvolved tissues (Fig. 2 A and Fig. S2 A). Gene set enrichment analysis (GSEA) comparing necrotic versus non-necrotic ROIs identified multiple pathways increased in necrotic ROIs related to neutrophil biology and lesion necrosis, including type-I IFN production and signaling, neutrophil activation/trafficking (chemotaxis, phagocytosis, reactive oxygen, and nitrogen species production), cell death, TGFβ signaling, and tissue degradation/remodeling (Fig. 2 B). Together, this suggests that even in primary infection settings, individual lesions have vastly different immune and inflammatory landscapes, with a dominant difference being type I IFN and neutrophil-associated factors.

To gain further insights into lesion development and effects of CoMtb on immune responses during infection, we performed single-cell RNA sequencing (scRNAseq) and flow cytometry analysis of lungs from Mtb-infected animals with and without CoMtb immediately preinfection and at 10, 17, and 34 days after CD infection. Clustering of scRNAseq data across time points and conditions allowed for robust identification of the major immune cell types comprising pulmonary lesions, including T cells, macrophages, neutrophils, and B cells (Fig. 2 C and Fig. S2 B). Primary infection of mice resulted in gradual recruitment of T cells, monocyte-derived cells (MDCs), as well as neutrophils, which continued to accumulate over time (Fig. 2, C and D). In contrast, infection of CoMtb mice induced an increased representation of activated CD4 T cells (primarily defined by *CD44* and *IFNg*) and MDCs at early time points (d17), and this was correlated with decreased bacterial burdens at this time point (Fig. 2 D and Fig. S2 C). The differences in CD4 T cells and MDC equalized by d34 (Fig. 2 D), when there was a greater increase in lung bacterial burdens in the primary group (Fig. S2 C). Neutrophils were found in equivalent representation at d10 and d17 for both conditions, but continued to increase in primary infected mice, while remaining stable in the CoMtb group (Fig. 2 D). Similar observations were confirmed by flow cytometry, demonstrating early increases in activated CD4 T cells and MDCs in CoMtb mice and increased neutrophil abundance in primary Mtb settings at later time points (Fig. 2 E; and Fig. S2, D and E).

To gain further insight into how CD4 T cells could be mediating immunity, we performed clustering analysis specifically on the activated CD4 T cell cluster (Fig. 2 F). Primary infection was associated with appearance of less differentiated T cells (aCD4.C1, typified by *Tcf7* and *Il7r*) on d17. This was followed by a large spike in multiple CD4 T cell populations on d34, including highly differentiated Th1 effector cells (aCD4.C0), which expressed elevated *Ifng* and *Gapdh*. CoMtb, on the other hand, was associated with the presence of Th1 effector cells (aCD4.C0) prior to infection, which led to a burst of both Th1 effector cells and sustained response from Th1/Th17 cells that have previously been shown to be correlated with protection in vaccination settings (aCD4.C2, expressing *Rora* and *Ifng*) (Dijkman et al., 2019; Darrah et al., 2023; Van Dis et al., 2018). This CoMtb-induced increase preceded the similar spike, which occurred at d34 during primary infection. Together, this suggests that the immunity afforded by CoMtb is associated with a reshaping of the earliest CD4 T cell response within the lung.

To characterize the response of neutrophils throughout infection, we performed pseudotime analysis to elucidate differences in cell populations, which do not always fall into discrete subsets (Grieshaber-Bouyer et al., 2021). For the first 10 days in both conditions, neutrophils expressed a transcriptional signature (“intermediate” [25–75] pseudotime) consistent with populations present at steady state, including cells expressing *Csf3r* and *Cxcr2* (Fig. 2 G and Fig. S2 F) (Hackert et al., 2023; Grieshaber-Bouyer et al., 2021). At d17, there was an emergence of a “low” (<25) pseudotime population in both groups, which was associated with decreased *Csf3r* and *Cxcr2*, as well as increase of *Fth1*, consistent with an increased activation state (Fig. 2 G and Fig. S2 F). At d34, there was a marked divergence, with the appearance of a high pseudotime population only in the primary mice, which coincided with the emergence of large necrotic lesions (Fig. 2 G). To better understand the factors responsible for this divergence, we performed clustering analysis of neutrophils at d34 (Fig. 2 H). This revealed that cluster N.C1, which comprised ∼30% of neutrophils in the primary setting and expressed factors reflecting neutrophil activation and recruitment (*CD300b*, *Bmx*, *Dck*, and *Ccl6*) (Garratt, 2021; Yamanishi et al., 2012; Takahashi et al., 2019), was significantly diminished in the setting of CoMtb. Further, N.C4, which expressed genes (*Lcn2*, *Ngp*, and *Camp*) suggestive of immature neutrophils, consistent with accelerated bone marrow egress in the setting of systemic infection (Grieshaber-Bouyer et al., 2021; Ganesh and Joshi, 2023), was completely extinguished in CoMtb. Both conditions exhibited similar numbers of N.C3, which likely represents a cluster undergoing cell death due to the predominance of mitochondrial genes. Together this indicates that CoMtb-mediated concomitant immunity results in pleiotropic effects on both innate and adaptive immune cell populations, with rapid recruitment of effector Th1 CD4 T cells into the infected lung and conversely by limiting the number of activated and immature neutrophils at later stages of the infection.

### CoMtb accelerates T cell and MDC activation, blunts neutrophil responses

**Figure 3.**
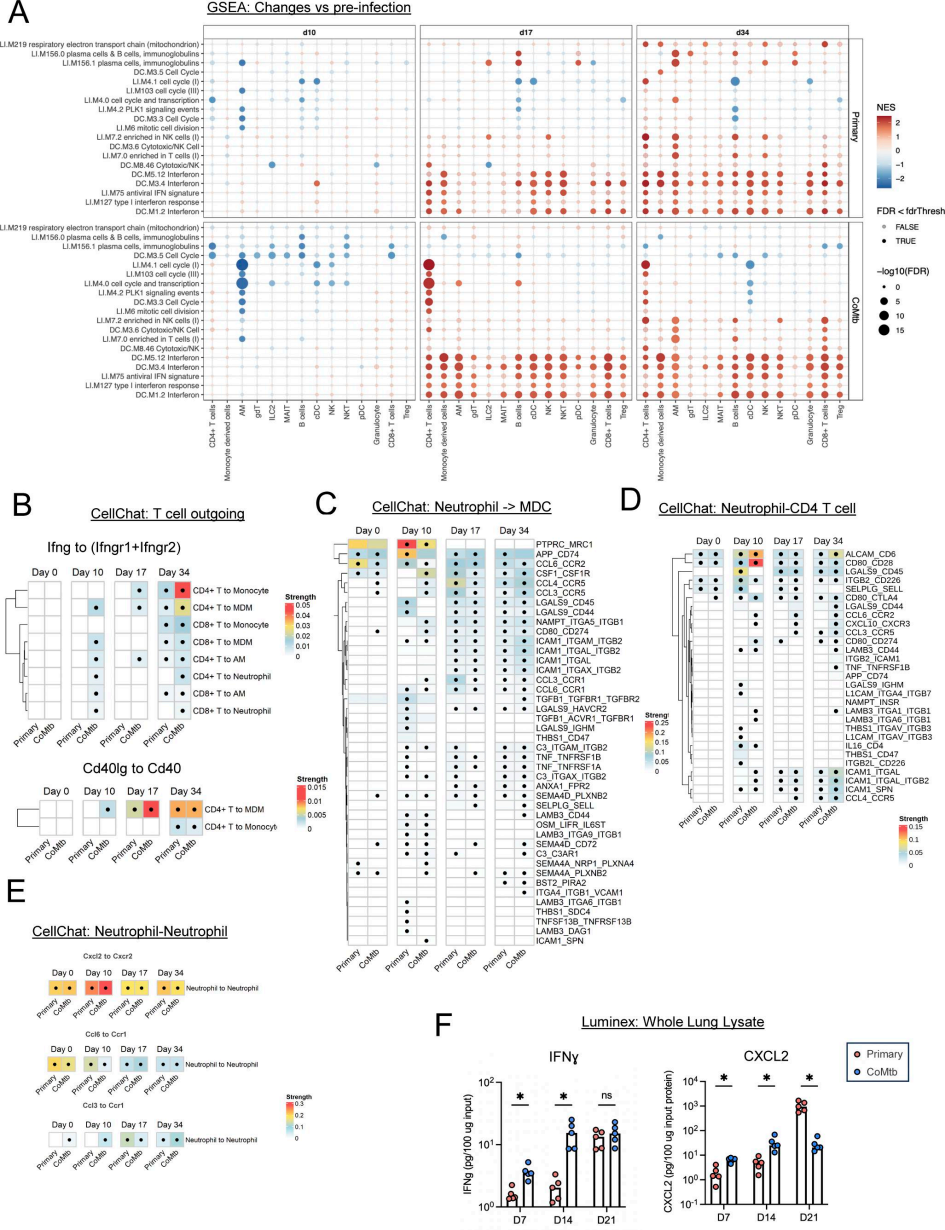
**CoMtb accelerates T cell and MDC activation, blunts neutrophil responses.** Multiple time points after CD infection. **(A)** GSEA analysis showing pathways enriched following aerosol infection at days 10, 17, and 34 p.i., in the setting of primary infection and CoMtb. **(B)** Predicted strength of selected T cell to myeloid cell signaling interactions quantified using CellChat. **(C)** Predicted strength of significant neutrophil to MDC interactions using CellChat. **(D)** Predicted strength of significant neutrophil to T cell interactions using CellChat. **(E)** Predicted strength of significant neutrophil to neutrophil chemotactic interactions using CellChat. **(F)** Levels of IFN and CXCL2, measured by Luminex. False discovery rate-adjusted P values determined using the R fgsea package. Dots in B–E indicate strength is significantly higher compared with a null distribution (i.e., CellChat-reported P value <0.05). *P < 0.05, and ns, P ≥ 0.05. Single-group comparisons in D and F by *t* test. Data are representative of one (A–E) or two independent experiments (F) experiments with at least four mice per group. See also Fig. S3. FDR, false discovery rate.

**Figure S3.**
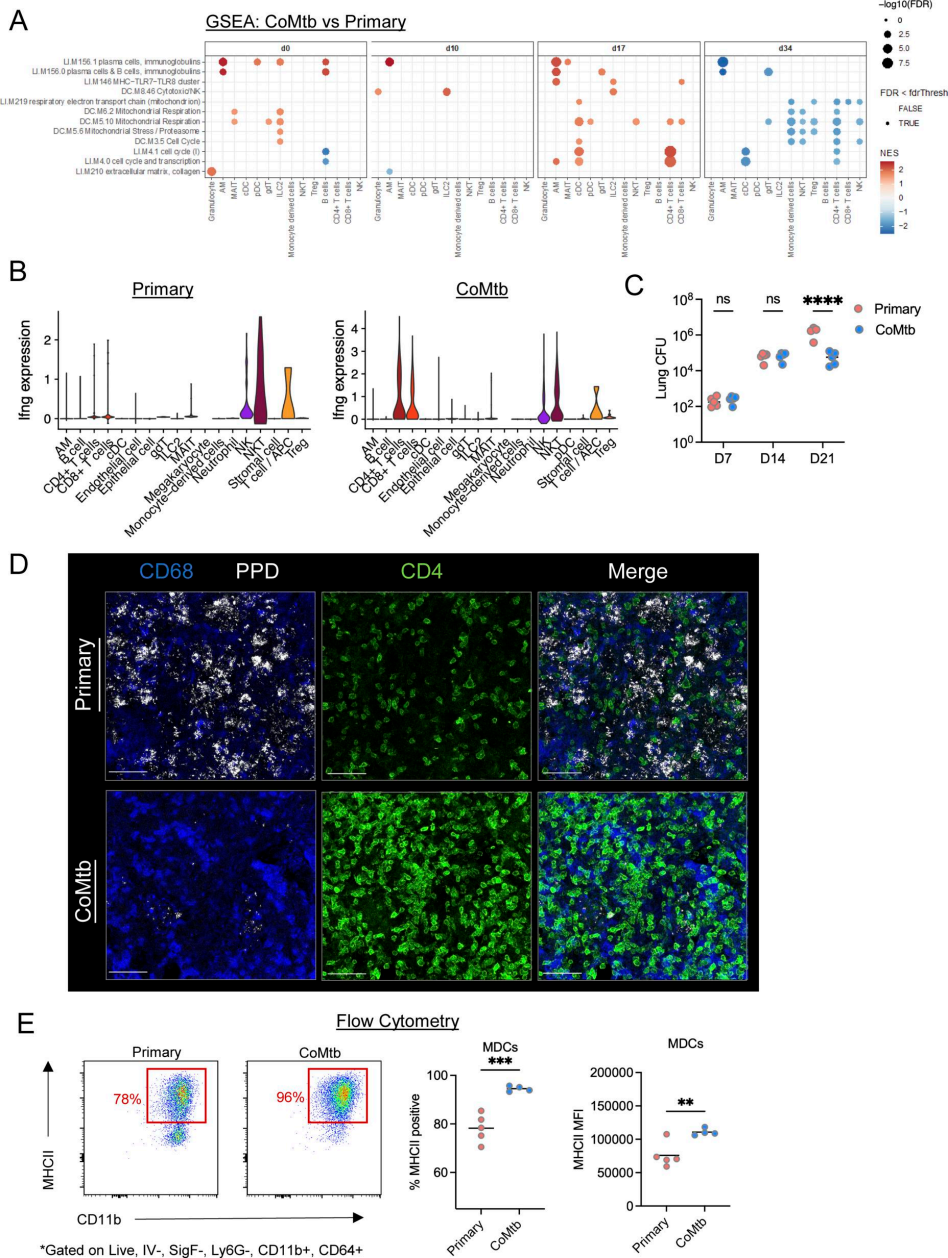
**CoMtb shapes early immune cell responses. (A–C)** Related to Fig. 3: CoMtb accelerates T cell and MDC activation, blunts neutrophil responses. **(A)** GSEA analysis comparing CoMtb to primary Mtb infection across time points, corresponding to Fig. 3 A. **(B)***Ifng* expression at d10 in our scRNAseq dataset, across all major cell types. **(C)** Pulmonary bacterial burdens corresponding to Fig. 3 F. FDR determined by the R fgsea package. Single-group comparisons by unpaired *t* test. **(D and E)** Related to Fig. 4. CoMtb shapes early tuberculous lesion cellularity and organization. **(D)** 1–2 color images from identical ROIs are shown in T cell panels in Fig. 4 A depicting density of CD4 T cells surrounding PPD in the lesions of primary and CoMtb mice. Scale bar: 50 μm. **(E)** Flow cytometry determination of the proportion and MFI of MHCII in MDCs, data from Fig. 2 E. Single-group comparisons by unpaired *t* test. **P < 0.01, ***P < 0.001, ****P < 0.0001, and ns, P ≧ 0.05. FDR, false discovery rate.

To further dissect the transcriptional changes in distinct cell types in the presence or absence of CoMtb over time, we used GSEA. Even prior to aerosol infection, there were differences in pathways associated with cell cycle and mitochondrial respiration in ILC2s in the setting of CoMtb, but not primary infection, potentially indicating innate training, which is consistent with previous reports (Fig. 3 A and Fig. S3 A) (Mai et al., 2024). In agreement with our cellular abundance analysis, a stronger cell cycle/division response was seen in CD4 T cells following CoMtb at d17, indicating increased cellular activation and proliferation of T cells within the lung parenchyma. Starting d17 p.i., we also observed striking response differences in IFN response pathways, which were upregulated over time across most cell populations (Fig. 3 A). Additional pathways upregulated at d17 in the CoMtb group included MHC-TLR7-TLR8 in alveolar macrophages (AMs) and NKT, cytotoxic/cell cycle in conventional dentritic cells (cDCs), and mitochondrial respiration in cDCs, plasmacytoid dendritic cells, natural killer (NK)Ts, CD4^+^, and CD8^+^ T cells. These changes, in turn, were associated with an increase in multiple pathways in the primary group at d34, including cell cycle in cDCs and mitochondrial respiration/stress in MDC, T cells, NK, and B cells.

To elucidate what might be driving these IFN pathways, we used CellChat analysis (Jin et al., 2021) to elucidate the predicted ligand–receptor interactions between T cells and myeloid cells, given that T cells were the only cell types to upregulate *Ifng* transcript at d10 following CoMtb (Fig. S3 B). We identified markedly increased outgoing *Ifng* signals from CD4 and CD8 T cells to multiple myeloid subsets in the setting of CoMtb at very early time points (d10) (Fig. 3 B), suggesting enhanced activation of myeloid cells by T cells. This was supported by Luminex analysis of whole-lung lysate, which demonstrated elevated levels of IFNɣ as early as d7 and d14 p.i. (Fig. 3 F and Fig. S3 C). Similarly, outgoing *Cd40lg* interactions from T cells to *Cd40* on myeloid cells were increased d10 and d17 p.i. (Fig. 3 B). Given that these time points are prior to (d10), or shortly after (d17), the time when changes in bacterial burdens are observed (Fig. S3 A), and that both IFNɣ and CD40L have important roles in Mtb immunity, these results suggest a role for T cell–derived activation of myeloid cells in CoMtb-mediated protection.

We also used CellChat to probe signaling between neutrophils and other immune cells. Given the opposing trajectories of neutrophils with T cells and MDCs over time, we first focused on outgoing signals from neutrophils to these cell types, finding that at d10 CoMtb increased costimulatory *Cd80–Cd28* interactions with T cells, while primary infection was associated with increased *Lgals9–Cd45* interactions with T cells and MDCs, which can be anti- or pro-inflammatory, depending on context, as well as likely inhibitory *App–Cd74* interactions with MDCs (Fig. 3, C and D) (Liu and Stowell, 2023; Rabinovich and Toscano, 2009). Next, we examined potential chemotactic interactions that could result in enhanced neutrophil recruitment, specifically focusing on neutrophil–neutrophil interactions that can drive feed-forward recruitment loops known to mediate tissue destruction in other models of inflammation (Kolaczkowska and Kubes, 2013). We found *Cxcl2–Cxcr2* interactions, which are known to drive such recruitment loops (Lentini et al., 2020), were increased in CoMtb mice at d10 but increased in primary infected animals at d34 (Fig. 3 E), which was associated with enhanced neutrophil abundance (Fig. 2, D and E). Similarly divergent CXCL2 protein abundance between the groups was also confirmed via Luminex. We found modestly increased CXCL2 protein abundance in settings of CoMtb at early time points (d14), but this was then dwarfed by massive upregulation of CXCL2 in primary infected animals 1 wk later (d21) (Fig. 3 F). Together, these data indicate that Mtb infection in CoMtb settings are associated with rapid recruitment and activation of T cells and enhanced IFNɣ sensing by local myeloid cells, as well as reprogramming of the neutrophil response, including limited recruitment at late time points. In contrast, primary infection in the absence of concomitant immunity is associated with limited early T cell activity and continued neutrophil influx over time.

### CoMtb shapes early tuberculous lesion cellularity and organization

**Figure 4.**
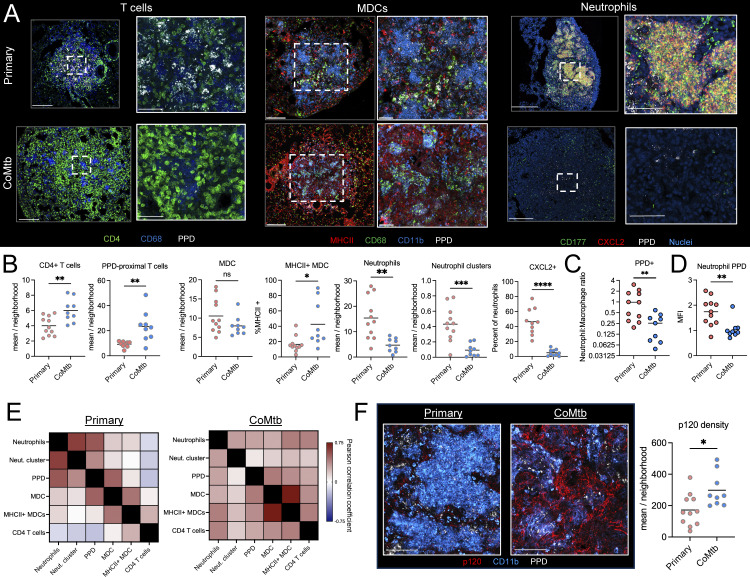
**CoMtb shapes early tuberculous lesion cellularity and organization.** d17 after CD infection, *n* = 5 per group. **(A)** Representative confocal images showing lesions and zoom-ins highlighting T cells, MDCs, and neutrophils. Scale bar: 200 μm; zoom: 50 μm. 1–2 color images from T cell regions are shown in Fig. S3 D. **(B)** Relative cellular density of specified cell types within lesions as determined by histo-cytometry. **(C)** Ratio of PPD+ neutrophils to PPD+ macrophages. **(D)** PPD MFI of PPD+ neutrophils. **(E)** Pearson correlation coefficients of the indicated cell populations within microenvironments. **(F)** Confocal image and spots/neighborhood of p120 staining. Scale bar: 50 μm. Single-group comparisons by unpaired *t* test. *P < 0.05, **P < 0.01, ***P < 0.001, ****P < 0.0001, and ns, P ≥ 0.05. Correlations by Pearson’s correlation test. Points represent individual lesions. Data are representative of two independent experiments with at least four mice per group. See also Fig. S3.

The above data indicated that prior Mtb exposure induces a fundamental shift in the pulmonary immune landscape during early Mtb lesion formation, which leads to divergent disease progression at later time points. To understand the organization of immune cells and investigate signaling microenvironments within developing lesions, we examined very early lesions at 17 days p.i. using quantitative microscopy. In accord with our observations by scRNAseq and flow cytometry, we observed accelerated CD4 T cell responses in CoMtb-infected mice, with a higher density of CD4 T cells within developing lesions, and particularly in neighborhoods in close proximity to PPD+ MDCs (Fig. 4, A and B; and Fig. S3 D). CoMtb mice also demonstrated an increased proportion of MHCII+ MDCs within lesions and increased proportion of MHCII and mean fluorescence intensity (MFI) by flow cytometry, consistent with increased coupling of T cell activation with downstream myeloid cell maturation (Fig. 4, A and B; and Fig. S3 E).

Many of the early lesions formed in the primary group already possessed extensive neutrophil clusters with robust CXCL2 expression, albeit we also observed extensive heterogeneity in this process, consistent with the divergent lesion outcomes seen at later time points (Fig. 4, A and B). In contrast, lesions formed in the setting of CoMtb possessed a much lower density of neutrophils, and the infiltrating cells were sparsely distributed throughout the lesions and did not express CXCL2 (Fig. 4, A and B). Neutrophils and MDCs represented relatively equal proportions of antigen-bearing cell types in primary mice, which is in contrast to CoMtb, which had a much lower proportion of antigen-bearing neutrophils (Fig. 4 C), which was accompanied by a lower PPD MFI within these cells, suggesting lower antigen burden (Fig. 4 D). The reduced neutrophil infiltration observed at this early time point by quantitative imaging differed markedly from the neutrophil cellularity observed at the same time point in flow cytometry and scRNAseq datasets, which showed similar neutrophil cellularity across groups, likely reflecting the inefficiency in recovering viable neutrophils in single-cell suspensions, especially when these cells are undergoing cell death, as seen in our spatial transcriptomics data (Fig. 2 B) (Ubags and Suratt, 2018). To further understand the spatial relationships of different cell types with respect to one another, we analyzed the cell–cell correlations of cellular abundance across tissue neighborhoods within lesions. We found that even at this early time point, there were already distinct organizational features. In primary mice without concomitant immunity, neutrophils were strongly associated with large clusters, as defined by aggregates >3,000 μm^3^, which were also highly associated with local PPD antigen abundance (Fig. 4 E), and both were negatively correlated with T cells. These lesions also showed evidence of decreased alveolar integrity as compared with CoMtb, with a decreased density of p120+ cells (Fig. 4 F). In contrast, CoMtb generated lesions in which PPD antigen was positively correlated with both macrophages expressing MHCII and CD4 T cells, suggesting closer proximity and cross talk between these cells. Together, this suggests that CoMtb has a dominant effect on shaping immune cell organization, abundance, activation, and permissiveness to infection within early developing pulmonary lesions following aerosol Mtb infection.

### CD4 T cells are required for CoMtb-mediated protection from lesion necrosis

**Figure 5.**
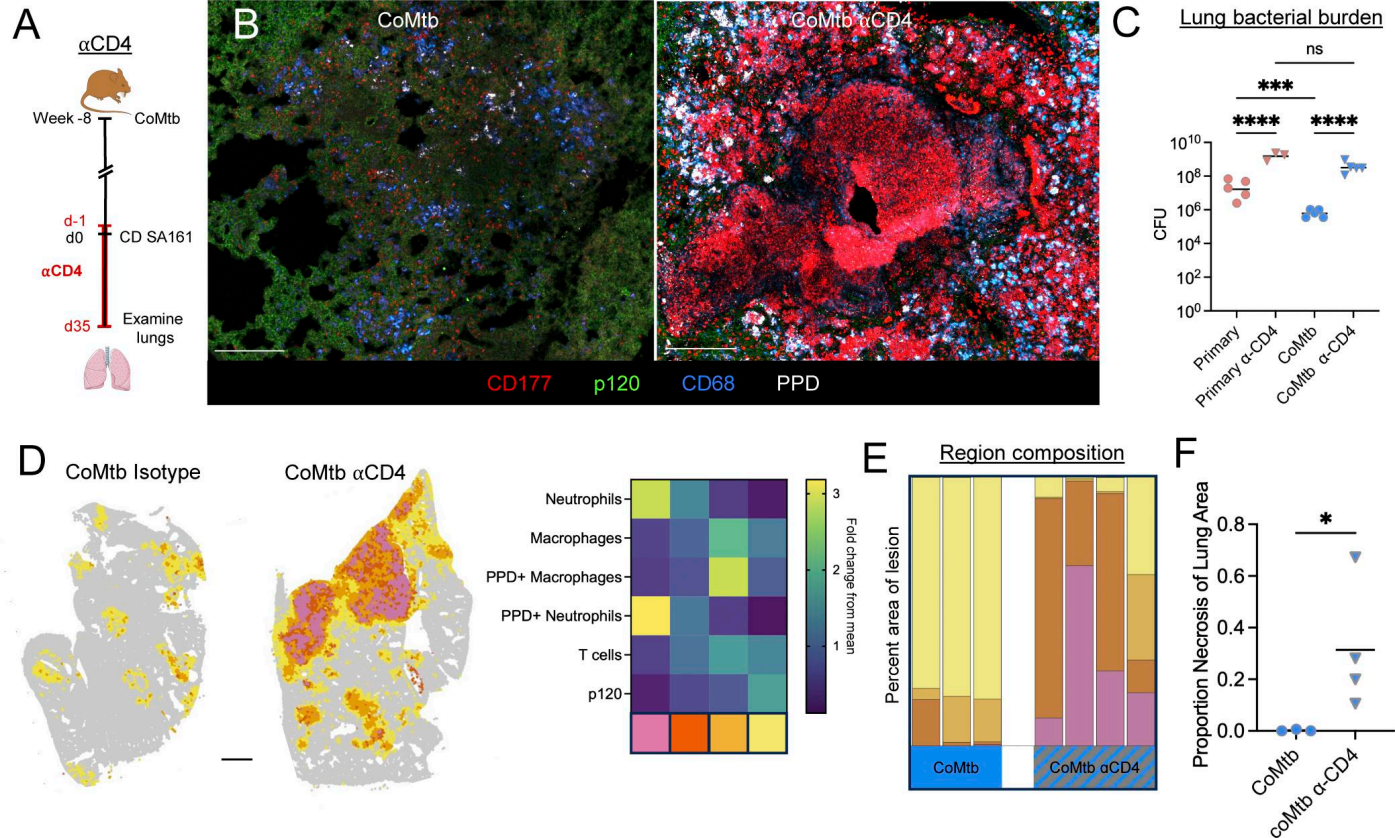
**CD4 T cells are required for CoMtb-mediated protection from lesion necrosis. (A)** Experimental outline. Subset of mice received CoMtb, then all mice aerosol infected with CD Mtb. Mice then received αCD4-depleting antibody or isotype from d-1 until harvest. *n* = 3–5 per group. **(B)** Representative confocal images showing presence of necrosis with αCD4 administration. Scale bar: 200 μm. **(C)** Pulmonary bacterial burdens. **(D)** Representative map showing 50 µm^2^ neighborhoods, color-coded microenvironment, and heatmap showing cellular composition of clustered microenvironments. Scale bar: 1 mm. **(E)** Percent area of lesion comprised by each microenvironment. Uninvolved regions (gray) not included. **(F)** Percent of lesion comprised by necrotic region (pink). Single-group comparisons by Mann–Whitney U test. *P < 0.05, ***P < 0.001, ****P < 0.0001, and ns, P ≥ 0.05. Points represent individual mice. Data are representative of two independent experiments with at least four mice per group. See also Fig. S4.

**Figure S4.**
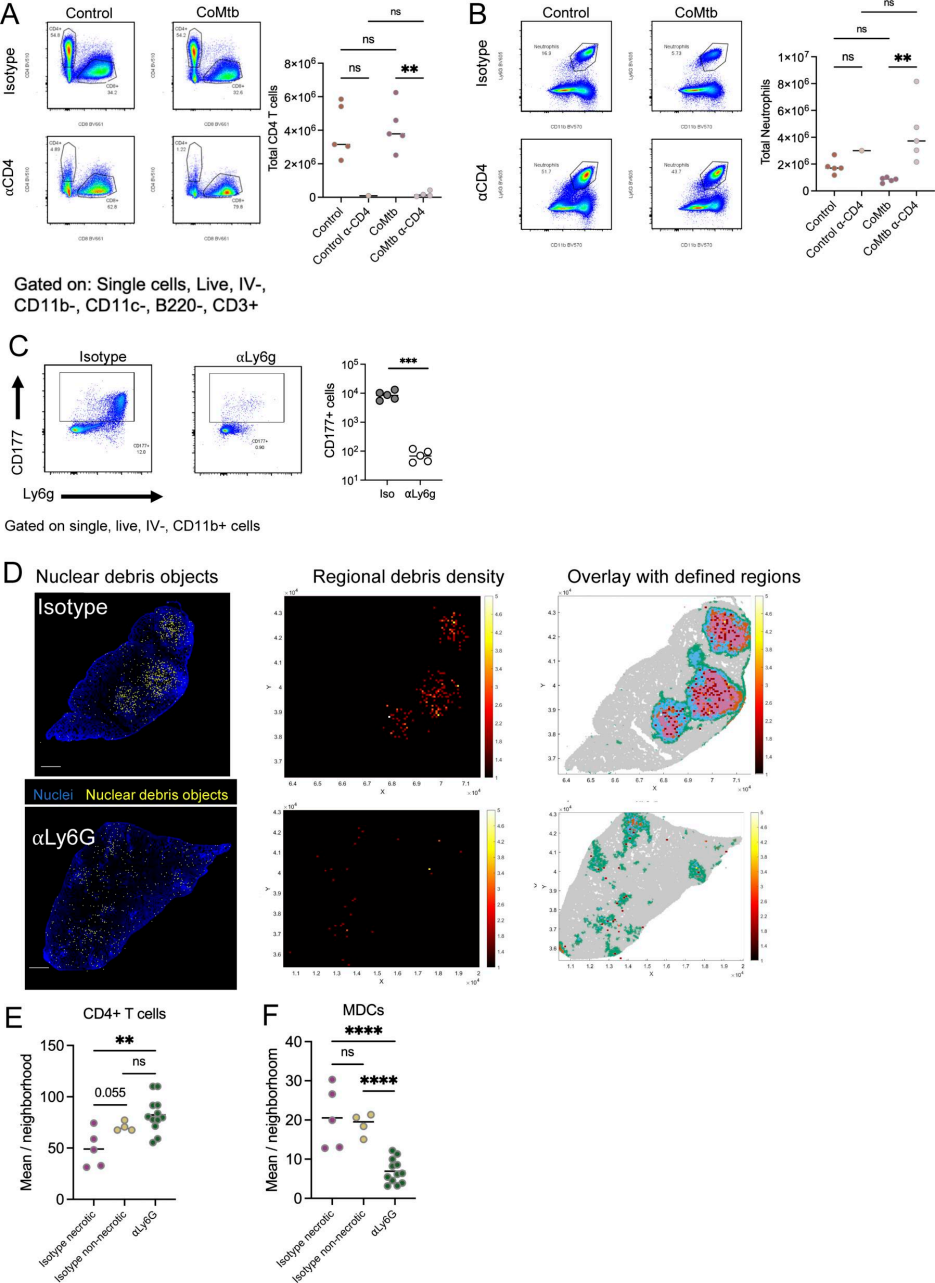
**CD4 T cell and neutrophil deplation shape lesion composition. (A and B)** Related to Fig. 5. CD4 T cells are required for CoMtb-mediated protection from lesion necrosis. **(A)** Representative flow plots and CD4 T cell enumeration following αCD4-depleting antibody administration. **(B)** Representative flow plots and neutrophil enumeration following αCD4-depleting antibody administration. Single-group comparisons by unpaired *t* test. **(C–F)** Related to Fig. 6: Neutrophils drive lesion necrosis. **(C)** Representative flow plots and neutrophil enumeration following αLy6G-depleting antibody administration. Also demonstrates concordance of CD177 and Ly6G in an Mtb-infected lung. **(D)** Validation of necrotic regions with nuclear debris objects. Scale bar: 1 mm. **(E)** Quantitative imaging determination of CD4^+^ T cell density within lesions. **(F)** Quantitative imaging determination of MDC density within lesions. Single-group comparisons by unpaired *t* test. **P < 0.01, ***P < 0.001, ****P < 0.0001, and ns, P ≧ 0.05.

We hypothesized that the CoMtb-mediated acceleration of CD4 T cell responses was responsible for improving local myeloid responses and CFU burden. To test this, we depleted CD4 T cells in primary and CoMtb-infected mice using anti-CD4 antibody, with the depletion beginning 1 day prior to infection, and examined lesion structures and lung CFU 35 days later (Fig. 5 A and Fig. S4 A). In stark contrast to aerosol Mtb-challenged CoMtb mice, which completely lacked necrotic lesions and possessed few, solitary neutrophils, CD4 T cell–depleted CoMtb mice developed highly necrotic lesions that contained a central core that was densely packed with infiltrating neutrophils and that lacked epithelial staining (Fig. 5, B and D–F; and Fig. S4 B). Depletion of CD4 T cells also led to a near-complete reversion of bacterial protection offered by CoMtb, resulting in minimal differences in lung CFU between primary and CoMtb-infected CD4-depleted mice (Fig. 5 C). Together, this suggests that CD4 T cells play a pivotal role in regulating neutrophil abundance/aggregation and lesion necrosis and are a major contributing factor in mediating the CoMtb reduction in bacterial burdens.

### Neutrophils drive lesion necrosis

**Figure 6.**
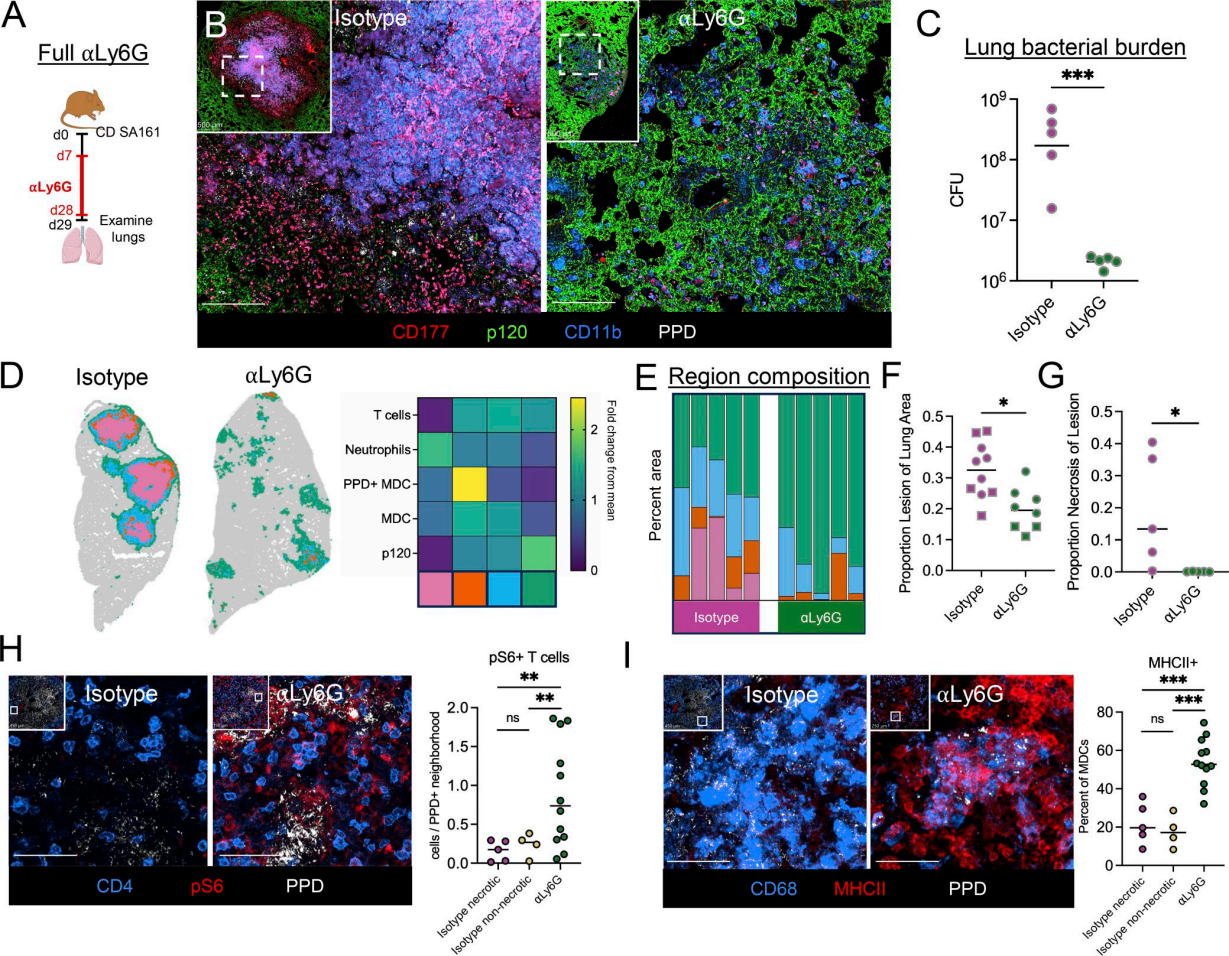
**Neutrophils drive lesion necrosis. (A)** Experimental outline for A–H. Mice received CD aerosol infection, then were administered αLy6G-depleting antibody or isotype from d7–d28, lungs were taken on d29. **(B)** Representative confocal images showing abrogation of necrosis with αLy6G administration. Scale bar: 150 μm. **(C)** Pulmonary bacterial burdens. **(D)** Representative map showing 50 µm^2^ neighborhoods, color-coded microenvironment, and heatmap showing cellular composition of clustered microenvironments Scale bar: 1 mm. **(E)** Percent area of lesion comprised by each microenvironment. Uninvolved regions (gray) not included. **(F)** Percent lesion (any color) of total lung area. **(G)** Percent of lesion comprised by necrotic region (pink). **(H)** Representative confocal images and quantification showing increased pS6+ T cells following αLy6G administration. Scale bar: 50 μm. **(I)** Representative confocal images and quantification showing increased MHCII+ in MDCs following αLy6G administration. Scale bar: 50 μm. Single-group comparisons by unpaired *t* test (C and F) or Mann–Whitney U test (G–I). *P < 0.05, **P < 0.01, ***P < 0.001, and ns, P ≥ 0.05. Points represent individual mice. Data are each representative of three independent experiments with at least four mice per group. See also Fig. S4.

Given the correlation between neutrophil abundance, increased bacterial burdens, and severe pathology that we and others have observed, we next hypothesized that neutrophils were necessary for pulmonary lesion necrosis and promote enhanced bacterial replication. The role of neutrophils during Mtb infection is multifaceted and contextual, with evidence suggesting both beneficial roles for bacterial control and detrimental roles driving worsened outcomes, especially in severe disease. Neutrophil depletion has been shown to compromise control of Mtb bacterial burden in the C3H model, though the impact of neutrophils on determining lesion organization has not been examined directly (Dallenga et al., 2017; Lovewell et al., 2021; Nwongbouwoh Muefong et al., 2022; Pedrosa et al., 2000). To directly test the role of neutrophils in promoting granuloma formation in the C3H mouse model, we infected mice with a CD of Mtb and depleted neutrophils with an anti-Ly6G (αLy6G and IA8) antibody starting at d7 p.i., when Mtb first starts to infect non-AM cell types, to d28 p.i., when mature necrotic lesions have formed (Fig. 6 A and Fig. S4 C). Neutrophil depletion resulted in a complete abrogation in lesion necrosis (Fig. 6, B and G) instead generating lesions comprised of alveolitis, akin to CoMtb, with less overall extent of lung involvement (Fig. 6, D–F). Given that neutrophils were a variable used in our region clustering algorithm (Fig. 6 D), we separately validated that these corresponded to areas with a high density of nuclear debris (Fig. S4 D). Neutrophil depletion also resulted in an approximately 2-log reduction in lung bacterial burdens (Fig. 6 C), together indicating that neutrophils promote lesion necrosis and restrict Mtb immune control.

To explore how neutrophils affect local immune landscapes within Mtb lesions, we again performed quantitative image analysis. Lesions in neutrophil-depleted mice exhibited enhanced infiltration of CD4 T cells into the Mtb-infected, macrophage-rich, central granuloma cores, including CD4 T cells with increased pS6 staining, suggesting recent TCR signaling, directly adjacent to PPD+ myeloid cells (Fig. 6 H and Fig. S4 E). Though the concentration of MDCs was significantly decreased by neutrophil depletion (Fig. S4 F), potentially secondary to decreased bacterial burdens, we observed markedly increased MHCII staining in neighboring cells, likely reflecting local inflammatory signaling and myeloid cell activation (Fig. 6 I).

### Neutrophils shape lesions throughout infection

**Figure 7.**
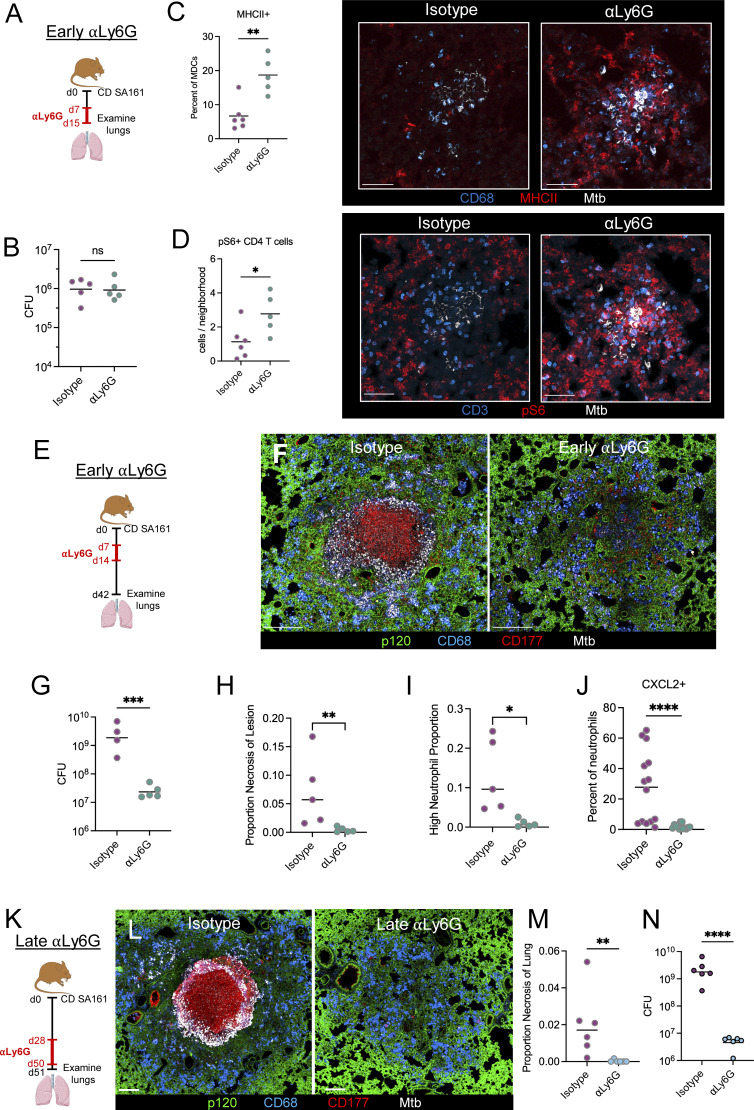
**Neutrophils shape lesions throughout infection. (A)** Experimental outline for B–D. Mice received CD aerosol infection, then were administered αLy6G-depleting antibody or isotype from d7–d15, lungs were taken on d15. **(B)** Pulmonary bacterial burdens. **(C)** Representative confocal images and quantification showing increased MHCII+ in MDCs following αLy6G administration. Scale bar: 50 μm. **(D)** Representative confocal images and quantification showing increased pS6+ T cells following αLy6G administration. Scale bar: 50 μm. **(E)** Experimental outline for F–I. Mice received CD aerosol infection, then were administered αLy6G-depleting antibody or isotype from d7–d15, lungs were taken on d43. **(F)** Representative confocal images showing abrogation of necrosis with “early” αLy6G administration. Scale bar: 250 μm. **(G)** Pulmonary bacterial burdens. **(H)** Percent of lesion comprised by necrotic region (pink). **(I)** Percent of lesion comprised by microenvironments with high neutrophil density. **(J)** Proportion of lesion neutrophils that express CXCL2. **(K)** Experimental outline for L–N. Mice received CD aerosol infection, then were administered αLy6G-depleting antibody or isotype from d28–d49, lungs were taken on d50. **(L)** Representative confocal images showing decreased necrosis with “late” αLy6G administration. Scale bar: 250 μm. **(M)** Percent of lung area comprised by necrotic region (pink). **(N)** Pulmonary bacterial burdens. Single-group comparisons by unpaired *t* test (B–D, G, and N) or Mann–Whitney U test (H–J and M). *P < 0.05, **P < 0.01, ***P < 0.001, ****P < 0.0001, and ns, P ≥ 0.05. Points represent individual mice. Data are each representative of two independent experiments with at least four mice per group. See also Fig. S5.

**Figure S5.**
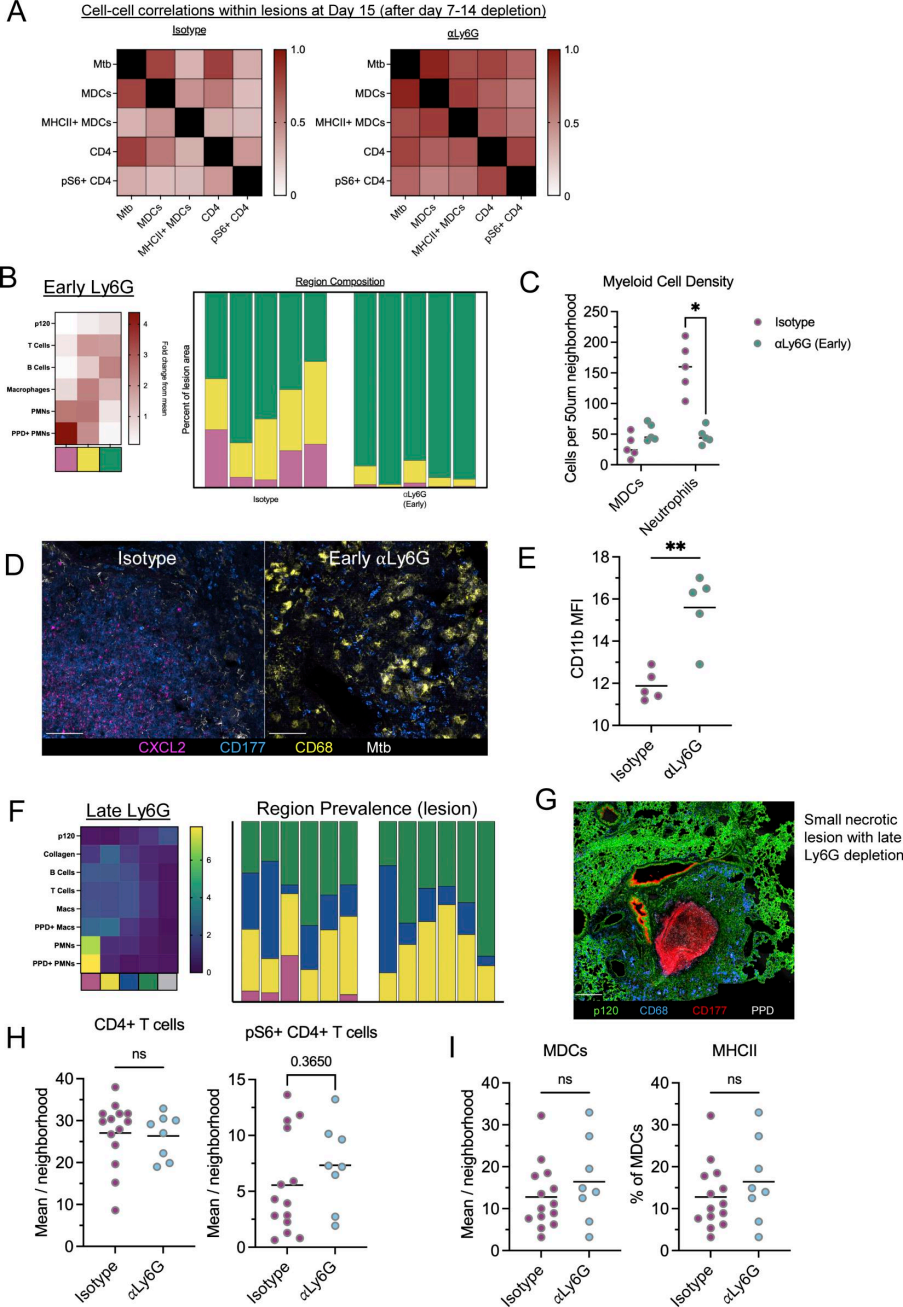
**Related to** Fig. 7 **.** Neutrophils shape lesions throughout infection. **(A)** Pearson correlation coefficients of the indicated cell populations within lesion microenvironments. **(B)** Heatmap showing cellular composition of clustered microenvironments and percent area of lesion comprised by each microenvironment, uninvolved regions not included, corresponding to Fig. 7 E. **(C)** Density of MDCs and neutrophils within lesions. **(D)** Representative confocal microscopy image showing CXCL2 staining (magenta). Scale bar: 50 μm. **(E)** Quantitative imaging determination of CD11b MFI within neutrophils. **(F)** Heatmap showing cellular composition of clustered microenvironments and percent area of lesion comprised by each microenvironment, uninvolved regions not included, corresponding to Fig. 7 J. **(G)** Confocal microscopy image of one small necrotic lesion with low antigen abundance, identified following late Ly6G depletion. Single-group comparisons by unpaired *t* test. Scale bar: 250 μm. **(H)** Quantitative imaging determination of CD4^+^ T cell and pS6+ CD4^+^ T cell density within lesions. **(I)** Quantitative imaging determination of MDC density and MHCII positivity within lesions. Single-group comparisons by unpaired *t* test. *P < 0.05, **P < 0.01, and ns, P ≧ 0.05.

We next hypothesized that neutrophil recruitment during the early stages of lesion formation might shape developing immune microenvironments and the downstream events of lesion progression. To test this, we administered ɑLy6G-depleting antibody 7–15 days p.i. (early depletion, Fig. 7 A) and then assessed responses within lesions immediately after treatment. Despite there being no difference in bacterial burdens between groups at d15 p.i. (Fig. 7 B), we found that Ly6G depletion resulted in an increased proportion of MHCII+ MDCs within developing lesions (Fig. 7 C). This increased myeloid activation was, in turn, correlated with an increase in the density of CD4^+^ T cells which had sensed antigen (as determined by pS6, Fig. 7 D). A correlation analysis of cellular abundance within lesion microenvironments revealed a strong correlation between Mtb bacilli, MHCII+ MDCs, and pS6+ CD4 T cell in neutrophil-depleted lesions, suggesting enhanced coupling of CD4-T cell/APC interactions in the absence of neutrophils even prior to bacterial burden differences (Fig. S5 A).

To assess for the effects of these changes on downstream infection outcomes, we administered αLy6G-depleting antibody 7–15 days p.i., then waited an additional 4 wk to allow for lesion development (Fig. 7 E). When we evaluated the lungs of these mice at d42, we observed that early neutrophil depletion completely blocked lesion necrosis, instead driving generation of alveolitis. Early neutrophil depletion also resulted in a 2-log decrease in lung bacterial burdens, comparable with that observed using the extended depletion protocol (Fig. 7, F–H and Fig. S5 B). Neutrophils were still observed in these lesions (Fig. S5 C), albeit at lower numbers as compared with full-depleted animals, but the cells that did infiltrate did not exhibit extensive clustering nor express CXCL2, and had a higher MFI for CD11b (Fig. 7, I and J; and Fig. S5, D and E). Together, this suggests that neutrophil influx during the early stages of lesion formation restricts CD4^+^ T cell–infected macrophage interactions, which subsequently drives necrosis granuloma development and downstream disease progression.

Finally, we examined whether continued neutrophil recruitment at later stages of infection, after the necrotic lesions have been established, is required for maintenance of disease pathology. To test this, we administered ɑLy6G antibody 28 days after CD infection with SA161 Mtb and continued treatment for 3 wk (late depletion) (Fig. 7 K). Evaluation of these lungs via imaging revealed that late neutrophil depletion also had marked beneficial effects on tissue pathology. Nearly all lesions in late-depleted animals lacked necrosis and caseation, and we found only a single lesion in 1/6 mice containing a small necrotic center (Fig. 7, L and M; and Fig. S5, F and G). Further, late neutrophil depletion was associated with a 2.5-log reduction in lung bacterial burdens (Fig. 7 N). No difference was observed in CD4^+^ T cell and MDC responses, likely confounded by differences in bacterial burdens (Fig. S5, H and I). Together, this suggests that continued neutrophil recruitment at late time points is required to propagate lesion necrosis and restrict immunity against Mtb.

## Discussion

From the earliest pathologic examinations of TB, it has been apparent that Mtb infection results in pulmonary lesions with vastly different organization, ranging from necrotizing and cavitating lesions to pneumonia-like alveolitis, and these pathologies are known to have major implications for disease severity and resiliency to antibiotic therapy (Hunter, 2020). Postmortem studies from the pre-antibiotic era showed an association between granuloma formation in primary TB and the development of alveolitis in individuals with prior immunity (Hunter, 2020; Hunter 2016; Rich, 1944), though the mechanistic basis for these associations and other potential reasons underlying lesion heterogeneity, remain unknown. Some historical studies examined differences between Mtb lesion structures in vaccinated versus unvaccinated animals (Henao-Tamayo et al., 2015; Ly et al., 2008), but lacked modern tools to examine cellular interactions in a quantitative manner and to perform mechanistic experiments. Conversely, most recent studies have focused on granuloma structures in animals in the absence of prior immunity, which limits lesion heterogeneity. Thus, very little is understood about the mechanisms driving different lesion structures, what microenvironments they possess, and how this is affected by pre-existing immunity. Using a mouse model of concomitant immunity (CoMtb) in C3H mice, we find that CoMtb completely abrogates the formation of necrotic granulomas and instead promotes the development of alveolitis, providing a tractable experimental system to assess how concomitant immunity shapes Mtb lesion structure. Our results support a model in which the relative kinetics of CD4 T cell versus neutrophil responses play major roles in governing the outcome of Mtb lesion formation. An early CD4 T cell response promotes immune control, prevents neutrophil dysregulation, and leads to alveolitis with intact lung epithelial architecture. In contrast, an early neutrophil response that precedes an adequate CD4 T cell response leads to a feed-forward cycle of neutrophil recruitment, which restricts the activity of late-arriving CD4 T cells, provides a permissive niche for Mtb replication, and drives tissue destruction and granuloma necrosis.

We observed neutrophils in Mtb lesions both during primary infection and in mice with CoMtb, but these neutrophils appeared to be in different functional states in each scenario. During primary infection, neutrophils were found in large aggregates in granuloma cores and expressed CXCL2, a chemokine known to promote feed-forward neutrophil recruitment and neutrophil swarming (Lentini et al., 2020), a phenomenon that has previously been associated with tissue damage (Kienle and Lämmermann, 2016; Brandes et al., 2013; Uderhardt et al., 2019; Peiseler and Kubes, 2019; Lämmermann et al., 2013). In contrast, in the setting of CoMtb, neutrophils did not aggregate or express CXCL2. The increased low-level CXCL2 that we observed in the lung homogenates of CoMtb mice at d7 and d14 could reflect production at early time points prior to a robust Mtb-specific T cell response, which at low levels distributed throughout the lung would be difficult to detect by the limited sensitivity and dynamic range of imaging, as well as production of CXCL2 by other cell types, such as macrophages or dendritic cells. Importantly, CD4 depletion in CoMtb mice completely reversed alveolitis formation and resulted in necrotic granulomas with large central aggregates of neutrophils expressing CXCL2, similar to primary granulomas observed without CoMtb. Studies in the rhesus model have shown that CD4 T cells act indirectly by reprogramming CD8 T cells and blunting type 1 signaling in myeloid cells, both of which could also impact neutrophil abundance (Bromley et al., 2024). Although CD4 T cells may indirectly impact neutrophil function by reducing lung bacterial burdens through their interactions with infected macrophages, our results suggest they may also regulate neutrophils more directly and that reduced bacterial burdens cannot completely explain our results. Our cell chat analysis revealed potential interactions between CD4 T cells and neutrophils through *Ifng*. IFNɣR signaling on neutrophils has been shown to induce neutrophil cell death, reduce neutrophil numbers, and even directly suppress CXCL2 expression (Thapa et al., 2013; Nandi and Behar, 2011; Stoolman et al., 2018). In addition, CD4 T cells may also act through other cell types, which in turn regulate neutrophil function. IFNɣR signaling on non-hematopoietic cells has been shown to be critical for neutrophil regulation during Mtb infection and may also play an important role in dictating the type of Mtb lesion formed (Blomgran and Ernst, 2011). Surprisingly, we also observed a relative paucity of differences in major transcriptional pathways when comparing effects of CoMtb in different cell types. This suggests that besides the specific identified inflammatory cross talk (e.g., *IFNɣ* in T cells and *Cxcl2* in neutrophils), early changes imparted by CoMtb are driven by changes in cellular recruitment and their function, and this in turn leads to major changes in tissue-level processes and disease progression. Although the critical role of CD4 T cells in controlling Mtb replication has been appreciated for years, the cellular and tissue-level processes by which CD4 T cells govern the formation of distinct Mtb lesion types is less understood and has been largely ignored. Future studies are needed to dissect the relative contributions of distinct mechanisms governing the regulatory axis between CD4 T cells and neutrophils in TB lesion formation and Mtb control.

Although a properly regulated neutrophil response has been shown to promote immunity against Mtb (Brown et al., 1987; Barrios-Payán et al., 2006; Sugawara et al., 2004; Martineau et al., 2007), and previous work has shown an association with neutrophils and neutrophil-driven processes such as NETosis with necrosis (Chowdhury et al., 2024; Moreira-Teixeira et al., 2020a), we show that a dysregulated and excessive neutrophil response is the primary mediator of tissue destruction in necrotic granulomas. Importantly, our results indicate that this tissue-destructive neutrophil response is not simply a response to poor Mtb control but is a key driver of the cascade of events that lead to adverse pathologic outcomes. We show that depletion of neutrophils at early stages of infection (d7–14) enhances myeloid and T cell activation at sites of infection even prior to the subsequent changes in bacterial burden and pathology. This suggests that an early neutrophil response that is unopposed by a sufficient CD4 T cell response can prevent late-arriving CD4 T cells from interacting with infected macrophages. Potential mechanisms identified in our studies include increased *App*–*Cd74* interactions from neutrophils to macrophages in primary infection, which has been shown to reduce phagocytic ability in tumor-associated macrophages (Ma et al., 2024). Additionally the increased *Lgals9* signals from neutrophils to T cells that we identified in primary infection could negatively regulate Th1 immunity via induction of cell death and suppression of cytokine expression (Liu and Stowell, 2023; Yang et al., 2021; Zhu et al., 2005; Rabinovich and Toscano, 2009). Importantly, 4 wk after administration of neutrophil-depleting antibodies was stopped 15 days p.i., a time point when there is no impact of neutrophil depletion on bacterial burdens, neutrophils rebounded and could be observed in the Mtb-infected lesions, but these neutrophils did not express CXCL2, aggregate, or lead to tissue damage. Furthermore, they exhibited increased expression of CD11b, a phenotype which is associated with resistance to Mtb replication (Lovewell et al., 2021). These findings demonstrate that there is a brief window for neutrophils to cause downstream lesion necrosis, which occurs prior to the divergence of bacterial burdens, and if neutrophils are not present during that time, responses by other cell types, such as those mediated by CD4 T cell and macrophage interactions, dominantly shape local tissue environments and alter downstream disease progression. Further studies are needed to see whether a sustained CD4 T cell response is needed to prevent necrosis. We also observed a dissociation between bacterial burdens and lung pathology during chronic stages of infection. Although BCG-immunized and CoMtb mice exhibited bacterial burdens similarly, or within a half-log, respectively, to mice with primary infection at d98 p.i., necrotic granulomas were completely abrogated in the former compared with the latter. Taken together, neutrophils begin to restrict immunity in primary infection even prior to differences in bacterial burdens compared with mice with prior or concomitant immunity, and furthermore, differences in bacterial burden are not the sole drivers of tissue pathology at later stages of infection.

In addition to their role in restricting CD4 T cell interactions with infected macrophages, our work, consistent with prior reports in the literature (Srivastava and Ernst, 2013), suggests that a dysregulated neutrophil response also contributes to adverse TB outcomes by providing a favorable intracellular niche for Mtb to reside. We find that early during infection, antigen-bearing neutrophils and macrophages are relatively equal in number, which is reduced by CoMtb, and at late time points, bacilli are present in large numbers in the neutrophil-rich necrotic core. The neutrophil core could provide residence for both extracellular and intracellular Mtb, and extracellular bacteria may cause epithelial cell damage to further skew lesions away from alveolitis (Saqib et al., 2025). The distinct metabolic state of neutrophils in primary infection compared with those in context CoMtb may contribute to their permissiveness to Mtb replication; our transcriptional analysis demonstrated the emergence of activated and immature neutrophil populations at d34 p.i., specifically during primary infection. The immature (N.C4) cluster, which was exclusively present in primary mice in this study, shared transcriptional similarities (*Camp*, *Ngp*, *Ltf*, and *Lcn2*) with those previously identified as being permissive to Mtb replication in B6 mice (Lovewell et al., 2021). These data support the emerging concept of neutrophil heterogeneity and that distinct subsets of neutrophils are induced during Mtb infection, which specifically drive pathological responses, and that pre-existing immunity prevents the induction of such responses. In addition, in most settings, neutrophils show a relatively poor ability to express MHCII (Vono et al., 2017). Because optimal Mtb control requires intrinsic MHCII expression on the infected cell (Srivastava and Ernst, 2013), this may be another factor that contributes to making neutrophils a conducive niche for Mtb to reside.

In addition to their role at early time points, we find that neutrophils are required for sustaining disease pathology and restricting immunity even after initial necrotic lesion formation, as depletion of neutrophils after formation of necrosis led to dramatic improvements in lesion pathology and marked reduction in lung bacterial burdens. This observation is consistent with the relatively short half-life of neutrophils, especially in inflamed tissues, and suggests that the detrimental outcomes associated with a dysregulated neutrophil response require ongoing neutrophil recruitment and function. To our knowledge, these improvements represent the greatest benefit observed after a host-directed intervention administered during late stages of Mtb disease. Although total neutrophil depletion is not a viable clinical strategy due to their importance in combating a wide range of infections, more targeted blockade of neutrophil recruitment, swarming, or function (such as NETosis [Moreira-Teixeira et al., 2020b; Kotov et al., 2023], type I IFN [Kotov et al., 2023], or ROS [Dallenga et al., 2017]) may be feasible, and deserves further study.

Consistent with historical observations in human postmortems, our observations in BCG-immunized and CoMtb mice provides direct experimental evidence that pre-existing immunity promotes Mtb lesions of alveolitis. However, our data also indicate that other factors can also influence Mtb lesion structure. During primary Mtb infection initiated by an infectious inoculum of ∼1 CFU, we observed that some mice developed necrotic granulomas, whereas others developed alveolitis, even in isogenic mice infected with genetically identical bacteria. These observations suggest that stochastic events, such as differences in the activation phenotype of the first cell to uptake Mtb or early cellular interactions in distinct regions of the lung, may also lead to necrotizing granulomas in some cases and alveolitis in others. Mtb strain characteristics and host genetics also likely contribute, as large necrotic granulomas form more readily in C3H mice infected with the Mtb SA161 strain than with the H37Rv strain (Moreira-Teixeira et al., 2020b), whereas infection by either Mtb strain in C57BL/6 mice, which mount a robust Th1 response, leads to lesions comprised of alveolitis even in the absence of prior immunity (Jung et al., 2009). Further evidence that genetic differences in the Mtb strains themselves can drive different lesion types comes from recent work showing that clinical Mtb strains associated with high transmission in human populations induce more granuloma necrosis in C3H mice than Mtb strains associated with low transmission (Verma et al., 2019). Environmental factors, including co-infections, may also shape Mtb lesion organization. Clinical studies demonstrate that HIV-infected patients with low CD4 T cell counts are less likely to form cavities (Kwan and Ernst, 2011). Initially, this seems incongruent with our findings that the absence of CD4 T cells strongly promotes necrosis. However, the CD4 depletion in HIV-infected individuals does not occur in isolation. These individuals are viremic, often have additional co-infections, and their immune systems are globally dysregulated, all of which may influence lesion structure (Bekker et al., 2023). Thus, while pre-existing immunity strongly influences lesion structure, additional work is needed to dissect the mechanisms driving lesion progression and disease pathogenesis across different settings and clinical scenarios.

One limitation of our work is that our studies were performed using a single inbred mouse strain (C3H) infected with a single Mtb strain (SA161). We chose this strain–strain combination with and without CoMtb because we found we could recapitulate the two basic lesions types (alveolitis versus necrotic granulomas), which in humans have been associated to occur with and without prior immunity, respectively. These pathologic outcomes are undoubtedly facilitated by the genetic background of the mouse; other genetic backgrounds can also lead to susceptibility and neutrophil-driven pathology (e.g., ACOD1^−/−^ and IFNgR^−/−^ mice) (Nandi and Behar, 2011; Nair et al., 2018). Furthermore, an excessive neutrophil response is strongly associated severe TB disease in mice, nonhuman primates, and humans (Lowe et al., 2013; Mattila et al., 2015; Niazi et al., 2015; Scott et al., 2020), but the genetic propensities that lead to severe TB susceptibility likely differ. This has led to the proposal of a “tipping point” model, which posits that a variety of host and environmental factors can lead to impaired host resistance, but each may trigger a common pathway of neutrophil-mediated disease exacerbation (Mayer-Barber, 2023). Future studies are needed to determine which aspects of CD4 and neutrophil regulatory circuits are unique to specific models versus generalizable to many forms of severe neutrophil-driven TB disease.

Overall, our work establishes a mouse model to dissect how concomitant immunity modifies disease progression and leads to the formation of distinct lesion types. Given the generation of necrotic lesions and alveolitis in relatively equal proportions in C3H mice infected with an inoculum of ∼1 CFU, this model is uniquely poised to uncover further mechanisms that tip the scales toward one lesion type or the other. Clinically, necrotic pulmonary lesions pose a significant challenge for antibiotic treatment, in large part due to the reduced penetration of antibiotics into necrotic centers. Necrotic lesions that have emptied their caseous core to form cavitary lesions also pose an increased risk of relapse and long-term pulmonary sequelae causing significant morbidity, such as impaired clearance of respiratory secretions and recurrent infections (Ravimohan et al., 2018; Benator et al., 2002). Our demonstration that neutrophil depletion, even administered after granuloma formation, can limit lung destruction and preserve alveolar epithelium architecture, suggests that neutrophils may provide a useful target for host-directed therapy in conjunction with antibiotic treatment. Recent studies in which positron emission tomography/computed tomographys were used to monitor TB patients undergoing therapy show that a subset of patients exhibited worse inflammatory changes upon initiation of antibiotics (Malherbe et al., 2020; Xie et al., 2021), likely as a result of the release of inflammatory bacterial products from dying bacteria. Multiple inhibitors of neutrophil trafficking and activation are currently developed for other indications and may be useful in reducing detrimental pathology and potentially even shortening treatment courses.

## Materials and methods

### Experimental model and subject details

#### Mice

C57BL/6 and C3H mice were purchased from Jackson Laboratories. All mice were housed in individually ventilated cages in specific pathogen–free conditions (maximum five mice/cage) within rooms with negative pressure ventilation and air filtering at Seattle Children’s Research Institute. Animals were monitored under care of full-time staff, given free access to food and water, and maintained under 12-h light and dark cycles, with temperature controlled between 22°C and 25°C. All possessed normal health and immune status. None had previous treatments, procedures, or invasive testing prior to the initiation of our studies. Experiments were approved by the Seattle Children’s Research Institute Animal Care and Use Committee and in adherence with the National Institutes of Health Guide for the Care and Use of Laboratory Animals. All experiments were conducted with sex- and age-matched mice (both male and female mice between the ages of 8–12 wk). The influence of sex was not assessed.

#### Mtb

For use in murine infections, Mtb SA161 strain was provided by Ian Orme (Colorado State University) (Palanisamy et al., 2009).

### Method details

#### Aerosol infections

Infections were done with a stock of Mtb SA161, as described previously (Urdahl et al., 2003). To perform CD aerosol infections, mice were placed in a Glas-Col aerosol infection chamber, and 50–100 CFUs were deposited into their lungs. To confirm the infectious inoculum, two mice per infection were euthanized on the same day of infection, then their lungs were homogenized and plated onto 7H10 or 7H11 plates for determination of CFUs. To perform ULD aerosol infections, mice were placed in a Glas-Col aerosol infection chamber, and 1–3 CFUs were deposited into their lungs (Plumlee et al., 2021).

#### CFU determination

Mouse organs (such as right or left lung, spleen) were individually homogenized in an M tube (Miltenyi) containing PBS+0.05% Tween-80. The resulting homogenates were diluted and plated onto 7H10 plates. Plates were incubated at 37°C for a minimum of 21 days before CFU enumeration.

#### Concomitant Mtb model (CoMtb)

The CoMtb model was established as described previously (Nemeth et al., 2020; Kupz et al., 2016). Briefly, mice were first anesthetized by intraperitoneal injection of 400 µl of ketamine (4.5 mg/ml) and xylazine (0.5 mg/ml) diluted in PBS. Mice were placed in a lateral recumbent position, and the ear pinna was flattened with forceps and pinned onto an elevated dissection board using a 22-G needle. H37Rv Mtb grown to an OD between 0.2 and 0.5 over a 48-h period was diluted to 10^6^ CFU/ml in PBS, and 10 μl (10^4^ CFU) was administered into the dermis of the ear using a 26s G Hamilton syringe. Mice were then rested for 6–8 wk prior to subsequent aerosol challenge.

#### Antibody depletions

For CD4 depletion studies, 500 μg of an anti-CD4–depleting antibody (clone GK1.5) was administered intraperitoneally to mice once weekly, from the day prior to aerosol infection until harvest. For neutrophil depletion studies, 200 µg of an anti-Ly6G–depleting antibody (clone IA8) was administered intraperitoneally to mice three times weekly for the specified time points.

#### Histology

Lungs processed for histology were fixed in 10% formalin for 24 h, then dehydrated in 70% ethanol at 4° for at least 24 h. Samples were paraffin embedded and sectioned at the University of Washington Histology Core. Subsequently, slides were reviewed by a veterinary pathologist and scored in a blinded fashion based on the following metrics (see Table S1): mixed granulomas (ill-formed granulomas with mixture of macrophages and lymphocytes), defined granulomas (well-defined with increased separation of macrophages, epithelioid or multinucleated giant cells with lymphoid aggregates), perivascular lymphoid aggregates, peribronchiolar lymphoid aggregates, histiocytes, foamy macrophages, multinucleated giant cells, alveolar hyperplasia, neutrophils, necrosis, cholesterol clefts, edema, extent 1 (percent involvement of the lung), and extent 2 (percent involvement of the lung in the worst manner).

#### Lung single-cell suspensions

At the indicated times p.i., mice were anesthetized with isoflurane and administered 1 μg anti-CD45.2 antibody intravenously. After 5–10 min of *in vivo* incubation, mice were euthanized by CO_2_ asphyxiation. Mouse lungs were excised and lightly homogenized in HEPES buffer containing Liberase Blendzyme 3 (70 μg/ml; Roche) and DNaseI (30 μg/ml; Sigma-Aldrich) using a gentleMacs dissociator (Miltenyi Biotec). The lungs were then incubated for 30 min at 37°C and then further homogenized a second time with the gentleMacs. The homogenates were filtered through a 70-μm cell strainer, pelleted for RBC lysis with RBC lysing buffer (Thermo Fisher Scientific), and resuspended in FACS buffer (PBS containing 2.5% FBS and 0.1% NaN_3_).

#### Antibody staining

Single-cell suspensions were first washed in PBS and then incubated with 50 μl Zombie UV viability dye (BioLegend) for 10 min at room temperature in the dark. Viability dye was immediately quenched by the addition of 100 μl of a surface antibody cocktail diluted in 50% FACS buffer/50% 24G2 Fc block buffer using saturating levels of antibodies. Surface staining was performed for 20 min at 4°C. Then, the cells were washed once with FACS buffer and fixed overnight with the eBioscience Intracellular Fixation and Permeabilization kit (Thermo Fisher Scientific). The following day, cells were permeabilized with the provided permeabilization buffer, incubated for 20 min at 4°C with 100 μl of an intracellular antibody cocktail diluted 1:100 in permeabilization buffer, and washed with FACS buffer. Cells were analyzed on a BD Symphony A5 cytometer (BD).

#### Antibodies

The following antibodies were used for staining mouse tissue sections for imaging or isolated cells for flow cytometry: B220 PCPCy5.5 (clone RA3-6B2; BioLegend), B220 PE/Fire 700 (clone RA3-6B2; BioLegend), CD103 PE (clone 2E7; BioLegend), CD105 R718 (clone MJ7/18; BD), CD11b BV480 (clone M1/70; BD), CD11b BV570 (clone M1/70; BioLegend), CD11b PCPCy5.5 (clone M1/70; BioLegend), CD11b PE/Fire 640 (clone M1/70; BioLegend), CD11b R718 (clone M1/70; BD), CD11c BV480 (clone HL3; BD), CD11c BV711 (clone N418; BioLegend), CD11c PCPCy5.5 (clone N418; BioLegend), CD11c PE (clone HL3; BD), CD177 AF647 (clone Y127; BD), CD177 CF555 (conjugated in-house) (clone Y127; BD), CD177 CF633 (conjugated in-house) (clone Y127; BD), CD177 PE (clone 1171A; R&D Systems), CD19 PE/Dazzle 594 (clone 6D5; BioLegend), CD26 PE-Cy7 (clone H194-112; BioLegend), CD3 BV480 (clone 17A2; BD), CD3 BV785 (clone 17A2; BioLegend), CD3 CF633 (conjugated in-house) (clone 17A2; BioLegend), CD3 PE/Fire 640 (clone 17A2; BioLegend), CD3 PE/Fire 700 (clone 17A2; BioLegend), CD3e BUV737 (clone 145-2C11; BD), CD4 BV510 (clone RM4-5; BioLegend), CD4 CF594 (conjugated in-house) (clone RM4-5; BioLegend), CD4 PE/Fire 700 (clone GK1.5; BioLegend), CD44 BV711 (clone IM7; BD), CD45.2 AF700 (clone 104; BioLegend), CD45.2 APC (clone 104; Thermo Fisher Scientific), CD45.2 R718 (clone 104; BD), CD62L AF488 (clone MEL-14; BioLegend), CD64 PerCP-eF 710 (clone X54-5/7.1; Thermo Fisher Scientific), CD68 BV421 (clone FA/11; BD), CD68 CF514 (conjugated in-house) (clone FA-11; Thermo Fisher Scientific), CD68 CF750 (conjugated in house) (clone FA-11; Thermo Fisher Scientific), CD69 PE/Dazzle 594 (clone H1.2F3; BioLegend), CD86 BUV737 (clone 2331 [FUN-1]; BD), CD8a BUV661 (clone 53-6.7; BD), Col1A1 CF660c (conjugated in-house) (clone E8F4L; Cell Signaling), Col1A1 CF750 (conjugated in-house) (clone E8F4L; Cell Signaling), CTLA4 (CD152) BV421 (clone UC10-4B9; BioLegend), CXCL2 CF555 (conjugated in-house) (polyclonal; R&D Systems), FoxP3 AF700 (clone FJK-16 s; Thermo Fisher Scientific), Gamma Delta TCR BUV805 (clone GL3; BD), iNOS AF405 (clone C-11; Santa Cruz Biotechnology), iNOS CF633 (conjugated in-house) (clone CXNFT; Thermo Fisher Scientific), Ki67 BV605 (clone 16A8; BioLegend), Ki67 BV650 (clone 11F6; BioLgend), Ki67 eF506 (clone SolA15; Thermo Fisher Scientific), KLRG1 BUV395 (clone 2F1; BD), Ly6C AF700 (clone HK1.4; BioLegend), Ly6G BV605 (clone 1A8; BioLegend), MHCII (I-Ab) FITC (clone KH74; BioLegend), MHCII (I-Ak) FITC (clone 10-3.6; BioLegend), MHCII AF700 (clone M5/114.15.2; BioLegend), MHCII BV480 (clone M5/114.15.2; BD), Mtb FITC (polyclonal; Abcam), NOS2 APC eF780 (clone CXNFT; Thermo Fisher Scientific), p120 AF488 (clone 6H11; Santa Cruz Biotechnology), p120 AF594 (clone 6H11; Santa Cruz Biotechnology), Phospho-S6 CF750 (conjugated in-house) (clone 2F9; Cell Signaling), Siglec F BV421 (clone E50-2440; BD), Siglec F BV480 (clone E50-2440; BD), SIRPα BV421 (clone P84; BD), and T-bet PE-Cy7 (clone 4B10; BioLegend).

#### Confocal microscopy

Lungs were removed and placed in BD Cytofix diluted 1:3 with PBS for 24 h at 4°C. Lungs were then washed two times in PBS and incubated in 30% sucrose for 24 h at 4°C. Lungs were then embedded in OCT and frozen in a dry ice slurry with 100% ethanol. A CM1950 cryostat (Leica) was used to generate 20-μm sections. Sections were rehydrated with 0.1 M Tris for 10 min, incubated for 1 h at room temperature with blocking buffer (0.1 M Tris with 1% normal mouse serum, 1% bovine serum albumin, and 0.3% Triton X100), and then stained for 6 h to overnight at room temperature with fluorescently conjugated antibodies. Following staining, slides were washed with 0.1 M Tris for 30 min and subsequently cover-slipped with Fluoromount G mounting media (SouthernBiotech). Images were acquired on a Leica Stellaris8 confocal microscope. For visual clarity, thresholds were applied to the displayed channel intensities in Imaris with identical settings applied across experimental groups.

#### Histo-cytometry

Histo-cytometry analysis was performed as described previously, with only minor modifications (Gerner et al., 2012). First, multiparameter confocal images were corrected for fluorophore spillover. Single color controls were made by mixing fluorophore-conjugated antibodies with Fluoromount G mounting media (SouthernBiotech) on a slide, then cover-slipping and collecting images with the same settings used for tissue imaging. Next fluorophore spillover was calculated and corrected using the Channel Dye Separation module in LAS X (Leica). Cell surfaces were created using Nucspot 750/780 nuclear staining using the Imaris surface creation module. Surfaces around neutrophils clusters were created on CD177 signal using the Imaris surface creation module (without splitting) followed by the application of size exclusion to only include surfaces >3,000 μm^3^. Nuclear debris surfaces were formed on nuclear signal, then filtered on objects with high ellipticity and low sphericity. The location of PPD and p120 signal was determined using the Imaris spot creation module. The surface object and spot statistics were exported as CSV files. Object statistics were concatenated into CSV files and imported into FlowJo software for hierarchical gating.

#### CytoMAP spatial organization analysis

Spatial organization analysis was performed using CytoMAP (Stoltzfus et al., 2020). In brief, the position of all cell objects within tissues was used for virtual raster scanning with 50-µm radius neighborhoods. Raster-scanned neighborhoods were also used for clustering based on cell type abundance (cell types used denoted in associate heat maps) to identify distinct region types, and these regions were used for heatmap and positional visualization of regions. For Fig. 4 B, cell-centered neighborhoods with 50-µm radius were created around PPD+ cells, and the T cell density within these regions was calculated. The Pearson correlation coefficient was calculated for the number of cells of the different cell types within these neighborhoods.

#### GeoMx DSP

A CM1950 cryostat (Leica) was used to generate 10-μm sections from lungs processed as outlined above, then stored at −80°C. During the sectioning process, lesions were classified as necrotic or non-necrotic by visual inspection (presence of caseum) and bright-field microscopy (assessing alveolar integrity and presence of necrotic debris). The fixed frozen sample slides were baked for 2 h at 60°C to ensure lung tissue adhered to slides. Following baking, we performed target retrieval for 20 min following all recommended settings (MAN-10115-04). RNA targets were exposed using recommended concentration and duration of proteinase K (1 µg/ml for 15 min). In situ probe hybridization took place overnight (18 h) using standard hybridization solution with no custom spike-in (v1.0) Mouse NGS Whole Transcriptome Atlas RNA - lot # MWTA12002). The next day, off-target probes were removed using stringent washes as recommended. Finally, morphology markers (SYTO13, B220 – PE, CD3 – CF594, and CD11b eF660) were added. Following antibody staining, slides and collection plate were loaded into GeoMx DSP instrument as recommended (MAN-10152-01). Slides were identified, and records were created for each.

Scan parameters were set for each channel: FITC/525 was utilized for SYTO13 nuclear staining, with exposure time of 50 ms. Cy3/568 nm was used for Alexa 532 to detect B220. Texas Red/615 nm was used for Alexa 594 to detect CD3. Cy5/666 nm was used for Cy5 to detect CD11b. All nonnuclear exposures were set for 200 ms. Configuration files were obtained from the nanostring website. SYTO13 was used for focus. Slides were then scanned. Multiple ROIs were obtained per lesion, including necrotic core (when applicable), inner lesion, outer lesion border, and full thickness (encompassing inner and outer areas), as assessed by nuclear density and autofluorescence pattern. More fine-grained region determination was not possible due to poor performance of antibody staining. ROIs were then collected.

#### GeoMx library prep and sequencing

Following collection, GeoMx samples were removed from the machine and allowed to air-dry overnight. The following day samples were placed in an open-top thermal cycler at 65°C for 10 min. Next, 10 µl of nuclease-free water were added to all samples well and pipetted up and down five times. PCR was run according to standard GeoMx protocols available in their quick start guide (MAN-10133-03). Pooling and cleanup were also run according to GeoMx protocols, with no deviations. The pooled library was assessed via Bioanalyzer and demonstrated a clean trace. Samples were loaded on the Illumina NextSeq platform at 1.6 pM and sequenced twice using the recommended paired-end 2 × 27 read acquisition. Sequenced library included 5% PhiX. Fastq files were assessed by QC metrics prior to further analysis.

#### GeoMx data analysis

Raw probe counts from 2 sequencing runs were combined at the fastq levels and then converted to .DCC files via Nanostring’s geomxngspipeline function. The DCC files were uploaded to the DSP instrument and automatically associated with individual scans. Sequencing and Probe quality control (QC) was performed using default parameters (Analysis suite version 2.5.1.145). 4 of 32 original samples were removed for low sequencing saturation.

Because we intended to pseudobulk ROIs within the same animal and thus required normalization strategies not available on the DSP analysis suite, we exported two datasets: (1) raw, post-QC Probe counts and (2) Q3-normalized counts (the recommended normalization approach by Nanostring).

For normalization assessment, we first removed control probes from the non-normalized data. These raw data were assessed by PCA, which indicated strong biases induced by raw reads, surface area, and nuclei count, as expected. Given the publication of some biases that occur when using the Q3 normalization strategy on GeoMx datasets, we used a compositionally aware normalization strategy known as a centered-log ratio approach. To obtain centered log-ratio (CLR)-transformed values for each gene, we first calculated the geometric mean of counts for each sample. We then created a ratio of an individual gene’s counts against the geometric mean from each sample. Finally, we calculated the log_2_ value of this ratio. Thus, all genes from a given sample were in essence normalized to their read depth. Unlike proportional (relative) normalization strategies, this method preserves the opportunity for downstream statistical analyses. We next assessed the samples using their CLR-transformed data by PCA. The CLR normalization strategy effectively eliminated the relationships between PCA dimensions and read depth, surface area, and nuclei count. Interestingly, when compared with PCA based on the Q3-normalized data (which also directly accounted for surface area and nuclei), the results were highly concordant. This contrasts sharply with recent accounts of Q3-induced skew in GeoMx datasets, which our assessments indicate were the result of using small, focused gene panels like the Cancer Transcriptome Atlas—and not a fundamental flaw in the Q3 strategy. We have reached similar conclusions when using targeted gene sets on the Nanostring nCounter. Because this PCA analysis appeared to validate our use of the compositionally aware CLR approach, all downstream data used CLR values.

To create a pseudobulked dataset, we first aggregated all raw, QC counts originally exported from the DSP, which created two sets of data per animal: aggregated counts from granuloma-associated ROIs and counts from distal, uninvolved regions. Due to the removal of samples for low-sequencing saturation (see above), 14/16 potential pseudobulk samples remained. Because CLR-transformed values are more appropriately assessed by Aitchison-distance PCoA (which is the Euclidean distance between CLR-transformed samples), PCoA was used for dimensionality reduction. To perform GSEA, we first calculated log2fc (using the CLR-transformed values) by directly comparing counts between necrotic and non-necrotic granulomas. We then used these log2fc values to rank genes. Ranked gene lists were supplied to a GSEA function in R, and results for significant enrichment and associated P values were obtained using the C5 ontology gene sets from MSigDB.

#### Luminex

For Luminex analyses, lungs from Mtb-infected mice were divided into three samples: the left lobe was homogenized in 1 ml PBS-Tween for CFU analysis, the inferior right lobe was placed in 5 ml Cytofix (BD) solution for overnight fixation and subsequent image analysis, and the remainder of the right lung was homogenized in 1 ml ProcartaPlex Cell Lysis Buffer (Thermo Fisher) supplemented with Halt Protease Inhibitor (Invitrogen) and DNaseI (30 μg/ml; Sigma-Aldrich) to generate protein lysates. After homogenization, the lysate was pelleted at maximum speed at 4°C for 10 min, and the supernatant was centrifuged through two sequential rounds of 0.2-μm SpinX (Costar) columns to sterilize the sample for removal from the BSL3 facility. Homogenates were then assayed for protein levels using a custom 17-plex ProcartaPlex kit following the manufacturer’s instructions (Luminex). Homogenates were also assayed for total protein content using a BCA assay (Pierce), and protein levels of each analyte were normalized to 100 ug protein input.

#### scRNAseq

Single-cell suspensions were generated from lung samples as described above prior to Mtb infection and at days 10, 17, and 34 after Mtb infection. Cells were resuspended in 200 μl MACS buffer (PBS containing 2.5% FBS plus 1 mM EDTA), filtered through a 70-μm filter, and run on a FACS AriaII (BD) sorter. To collect parenchymal cells for scRNAseq, AMs (SiglecF+CD11c+) were sorted separately into one collection tube to account for autofluorescence in the IV label channel, and all other IV-negative cells were sorted into another collection tube. After sorting, the two populations were combined and counted on a hemocytometer. After one round of washing with ice-cold DPBS, cells were resuspended to 1,000 cells/μl in DPBS, and 8,000 cells were inputted into the 10X Genomics pipeline following the manufacturer’s recommendations. After the generation of cDNA following the manufacturer’s protocol, samples were centrifuged through two sequential rounds of 0.2-μm SpinX (Costar) columns to sterilize the sample for removal from the BSL3 facility and subsequent library generation. Libraries were submitted to Psomagen for NovaSeq sequencing, with 300 M reads per sample.

#### Alignment and processing of scRNAseq data

10X chromium 3′-derived scRNAseq sequence reads were aligned to the 10X Genomics pre-built mouse reference genome mm10-2020-A, assigned to individual cells by barcode, and unique molecular identifiers (UMI) summarized using the 10X Cell Ranger 7.1.0 software package.

The Seurat R package was used for initial QC filtering and integration. First, a filtering step was applied across all samples, requiring all passing cells to have UMIs mapped to at least 500 distinct genes and fewer than 5% of UMIs mapped to mitochondrial genes. Genes detected in fewer than three cells per mouse were excluded from further analysis. The Seurat integration pipeline (Butler et al., 2018) was then applied to correct for batch effects and align cells across conditions, including all combinations of mouse strain, Mtb strain, time after challenge, and CoMtb status.

Initial cell type assignment was performed using the CellTypist python package (Domínguez Conde et al., 2022). As CellTypist does not have an available cell type model suitable for mouse lung or mouse immune cells, we created a de novo mouse lung immune cell type model using two published mouse cell atlases, namely the Tabula Muris (Tabula Muris Consortium et al., 2018) and scMCA (Han et al., 2018) resources. Cell type labels were harmonized between both sources (e.g., macrophage -> Macrophage), and both datasets were filtered to retain immune and lung-associated cell types, excluding cells specific to other organs. The CellTypist python package was then used to train a mouse lung cell type model based on this combined resource. This model was then used to assign cell types to count normalized log-transformed data on a per cell level from mouse lung scRNAseq samples, using the python scanpy (Wolf et al., 2018) package to normalize total counts per cell to 10,000 and log transform as required by CellTypist.

After initial cell type labeling, further unsupervised clustering of specific cell subtypes was performed for cells labeled as “T cells” or “NK cells” and separately for all APC subtypes, i.e., “AM,” “Dendritic cell,” “Monocyte,” and “Macrophage.” Unsupervised clustering was run using the standard Seurat pipeline, which identifies the top 2,000 most variable genes in the data, creates a shared nearest neighbor network of cells, and divides the nearest neighbor network into discrete clusters using the Louvain algorithm. The resulting clusters were manually annotated by identifying differentially expressed marker genes for each cluster (using the Seurat FindAllMarkers) function, and these marker genes were linked to known cell types, e.g., Cd4^+^ IFNg^+^ Th1 cells express high levels of Cd3, Cd4 and Ifng.

To quantify changes in cell type proportion over time, total numbers of cells per sample were calculated and normalized to cells per thousand per sample. Negative-binomial linear models, appropriate for zero-inflated count data, were fit and used to calculate P values using the R glm.nb function.

Gene expression changes within specific cell types were determined using a pseudobulk approach, where counts from all similarly labeled cells were combined into a single sample x gene count matrix using the Seurat AggregateExpression function. The standard bulk RNAseq analysis package DESeq2 (Love et al., 2014) was then used to calculate differential expression fold-changes and P values for contrasts of interest.

Ranked gene lists from the above pseudobulk analysis were used as input for GSEA using the R fgsea package (Korotkevich et al., 2021, *Preprint*). Gene sets used were previously published human coherent blood transcriptional modules (Obermoser et al., 2013; Li et al., 2014) as available in the R tmod (Zyla et al., 2019) package, as well as mechanistic pathway modules from the Reactome database (Fabregat et al., 2018) as available in the R msigdbr package (Liberzon et al., 2011). To adapt human blood transcriptional gene sets to mouse, human genes were mapped to mouse orthologs using the Jackson Lab Mouse Genome Informatics Human-Mouse mapping (https://www.informatics.jax.org/downloads/reports/HOM_MouseHumanSequence.rpt). The resulting mouse-translated gene sets were filtered to retain only blood transcriptional modules with at least five mouse genes, where >80% of the original human genes were successfully mapped to mouse orthologs. Unannotated gene sets (“TBA” or “Undetermined”) were removed from further analysis.

We used the CellChat analysis package (Jin et al., 2021) (version 1.6.1) to quantify the strength of receptor–ligand communications among cell types in our scRNAseq dataset. To simplify interpretability and ensure a sufficient number of cells of each type in the analysis, the sub-types of CD4^+^ and CD8^+^ T cells were grouped into the broader categories “CD4^+^ T cell” and “CD8^+^ T cell.” Additionally, the IM and monocyte sub-types were grouped into “MDCs,” and AM sub-types were grouped into a broader “AM” category. Our analyses focused on examining (1) whether intercellular communications originating with T cells and targeting myeloid cells differed between the primary and CoMtb conditions and (2) whether neutrophil-to-neutrophil chemotactic communications (those represented in the “CCL” and “CXCL” CellChat pathways) differed between conditions.

#### Subclustering activated CD4^+^ T cells

Subclustering of activated CD4^+^ T cells was performed by rerunning steps of the R Seurat (Hao et al., 2024) clustering pipeline tailored specifically to the activated CD4^+^ T cell subset. Firstly, the top 2,000 most variable activated CD4^+^ T cell genes were identified, and then the PCA transform was recomputed. Subclusters were then identified using the FindNeighbors and FindCluster functions run with a resolution of 0.2, using all 30 PCA coordinates. Cell counts per subcluster per sample normalized per thousand total immune cells were calculated, and subcluster-specific marker genes were identified using the Seurat FindAllMarkers function with the ROC test.

#### Neutrophil pseudotime analysis

All neutrophil-annotated cells were used as input to pseudotime analysis, which was performed using the R slingshot package (Street et al., 2018). Initially, the top 2,000 most variable genes within neutrophils were identified, expression levels were scaled and centered, and PCA transforms were calculated using the R Seurat pipeline (Hao et al., 2024). Model-based Gaussian mixture modeling was performed, using the R Mclust package using the top 10 principal components. The top 10 principal components and Gaussian mixture modeling clusters were used as input to slingshot pseudotime trajectory inference function, which produced numerical pseudotime estimates per cell. Pseudotime values were categorized into low pseudotime <25;=, intermediate 25 < pseudotime <75, and high pseudotime >75 for plotting and differential expression comparisons. P values for differentially expressed genes between pseudotime categories were identified using false discovery rate–adjusted *t* tests. GSEA was performed using the mSigDB Hallmark gene sets (Liberzon et al., 2015).

#### Subclustering neutrophils

Subclustering of neutrophils was performed by rerunning steps of the R Seurat (Hao et al., 2024) clustering pipeline tailored specifically to the neutrophil cell cluster. Firstly, the top 2,000 most variable neutrophil genes were identified, and then the PCA transform recomputed. Subclusters were then identified using the FindNeighbors and FindCluster functions run with a resolution of 0.2, using all 30 PCA coordinates. Cell counts per subcluster per sample normalized per thousand total immune cells were calculated, and subcluster-specific marker genes were identified using the Seurat FindAllMarkers function with the ROC test.

#### Quantification and statistical analysis

Statistical tests were selected based on appropriate assumptions with respect to data distribution and variance characteristics. Statistical details of experiments can be found in the figure legends. No statistical methods were used to predetermine sample size. The statistical significance of differences in mean values was determined by the appropriate test, as denoted in the figure legends. Paired *t* tests were performed only when comparing responses within the same experimental animal or tissue or group means within the same experiment (indicated in the legend). Correlations and corresponding P values by Pearson’s correlation test. ∗∗∗∗, P ≤ 0.0001; ∗∗∗, P ≤ 0.001; ∗∗, P ≤ 0.01; ∗, P ≤ 0.05; and ns, P > 0.05.

### Online supplemental material

Fig. S1 shows the organizational composition of primary and CoMtb lesions. Fig. S2 shows the broad transcriptional changes enacted in necrotic lesions and with CoMtb. Fig. S3 shows the early transcriptional programs and cell–cell interactions affected by CoMtb. Fig. S4 shows the data supporting the analysis of CD4 T cell and neutrophil depletions. Fig. S5 shows the changes in lesion microenvironments, which occur during neutrophil depletion. Table S1 shows the lung pathology scoring of control, BCG-immunized, and CoMtb mice.

## Supplementary Material

Table S1 shows that the pre-existing immunity abrogates the formation of necrotic granulomas.

## Data Availability

Data are available in the article itself and its supplementary materials. The RNAseq data underlying Figs. 2, S2, 3, and S3 are openly available at the Gene Expression Omnibus under accession nos. GSE264267 and GSE265843.
