# Supplementary material for: Early and opposing neutrophil and CD4 T cell responses shape pulmonary tuberculosis pathology
Source: J Exp Med. 2025 Jul 30;222(10):e20250161. doi: 10.1084/jem.20250161 (PMC12309470; doi:10.1084/jem.20250161)
Supplement: Table S1 — shows that the pre-existing immunity abrogates the formation of necrotic granulomas. [file jem_20250161_tables1.docx]

|  | **Extent 1** | **Extent 2** | **Mixed Granulomas** | **Defined Granulomas** | **Perivasclar Lymphoid Aggregates** | **Peribroncial Lymphoid Aggregates** | **Histiocytes** | **Foamy Macrophages** | **Multinucleated Giant Cell** | **Neutrophils** | **Necrosis** | **Cholesterol Clefts** |
| --- | --- | --- | --- | --- | --- | --- | --- | --- | --- | --- | --- | --- |
| **Primary 1** | 3 | 3 | 1 | 4 | 2 | 1 | 3 | 3 | 2 | 2 | 3 | 2 |
| **Primary 2** | 3 | 2 | 2 | 2 | 2 | 1 | 3 | 3 | 2 | 3 | 1 | 2 |
| **Primary 3** | 3 | 3 | 1 | 3 | 2 | 1 | 3 | 3 | 1 | 2 | 3 | 2 |
| **Primary 4** | 3 | 2 | 1 | 3 | 2 | 0 | 3 | 3 | 1 | 2 | 3 | 2 |
| **Primary 5** | 4 | 3 | 1 | 4 | 3 | 1 | 3 | 3 | 2 | 2 | 4 | 2 |
| **BCG 1** | 3 | 2 | 1 | 2 | 2 | 1 | 3 | 4 | 1 | 2 | 1 | 2 |
| **BCG 2** | 2 | 2 | 2 | 2 | 2 | 1 | 3 | 3 | 1 | 2 | 1 | 2 |
| **BCG 3** | 3 | 2 | 2 | 2 | 1 | 1 | 3 | 3 | 2 | 2 | 1 | 2 |
| **BCG 4** | 2 | 2 | 1 | 2 | 1 | 1 | 2 | 2 | 1 | 1 | 1 | 2 |
| **BCG 5** | 3 | 2 | 1 | 3 | 1 | 1 | 3 | 3 | 1 | 2 | 1 | 2 |
| **CoMtb 1** | 2 | 1 | 0 | 1 | 1 | 1 | 1 | 1 | 0 | 1 | 0 | 0 |
| **CoMtb 2** | 2 | 1 | 2 | 1 | 1 | 1 | 2 | 2 | 1 | 0 | 1 | 1 |
| **CoMtb 3** | 3 | 1 | 1 | 1 | 3 | 1 | 1 | 1 | 0 | 1 | 0 | 0 |
| **CoMtb 4** | 2 | 2 | 0 | 3 | 2 | 1 | 2 | 3 | 0 | 1 | 0 | 1 |
| **CoMtb 5** | 2 | 2 | 1 | 1 | 1 | 1 | 2 | 2 | 0 | 1 | 0 | 0 |

**Table S1. Pre-existing immunity abrogates the formation of necrotic granulomas. Related to Fig. 1.** Pathology scores for hematoxylin and eosin-stained tissue sections in Fig. 1 A.
